# Basic Principles of Emulsion Templating and Its Use as an Emerging Manufacturing Method of Tissue Engineering Scaffolds

**DOI:** 10.3389/fbioe.2020.00875

**Published:** 2020-08-12

**Authors:** Betül Aldemir Dikici, Frederik Claeyssens

**Affiliations:** ^1^Department of Materials Science and Engineering, Kroto Research Institute, The University of Sheffield, Sheffield, United Kingdom; ^2^Department of Materials Science and Engineering, INSIGNEO Institute for In Silico Medicine, The University of Sheffield, Sheffield, United Kingdom

**Keywords:** emulsion templating, tissue engineering, biomaterials, scaffold, PolyHIPE, porosity, interconnectivity, tunability

## Abstract

Tissue engineering (TE) aims to regenerate critical size defects, which cannot heal naturally, by using highly porous matrices called TE scaffolds made of biocompatible and biodegradable materials. There are various manufacturing techniques commonly used to fabricate TE scaffolds. However, in most cases, they do not provide materials with a highly interconnected pore design. Thus, emulsion templating is a promising and convenient route for the fabrication of matrices with up to 99% porosity and high interconnectivity. These matrices have been used for various application areas for decades. Although this polymer structuring technique is older than TE itself, the use of polymerised internal phase emulsions (PolyHIPEs) in TE is relatively new compared to other scaffold manufacturing techniques. It is likely because it requires a multidisciplinary background including materials science, chemistry and TE although producing emulsion templated scaffolds is practically simple. To date, a number of excellent reviews on emulsion templating have been published by the pioneers in this field in order to explain the chemistry behind this technique and potential areas of use of the emulsion templated structures. This particular review focusses on the key points of how emulsion templated scaffolds can be fabricated for different TE applications. Accordingly, we first explain the basics of emulsion templating and characteristics of PolyHIPE scaffolds. Then, we discuss the role of each ingredient in the emulsion and the impact of the compositional changes and process conditions on the characteristics of PolyHIPEs. Afterward, current fabrication methods of biocompatible PolyHIPE scaffolds and polymerisation routes are detailed, and the functionalisation strategies that can be used to improve the biological activity of PolyHIPE scaffolds are discussed. Finally, the applications of PolyHIPEs on soft and hard TE as well as *in vitro* models and drug delivery in the literature are summarised.

## Introduction

**FIGURE 1:**
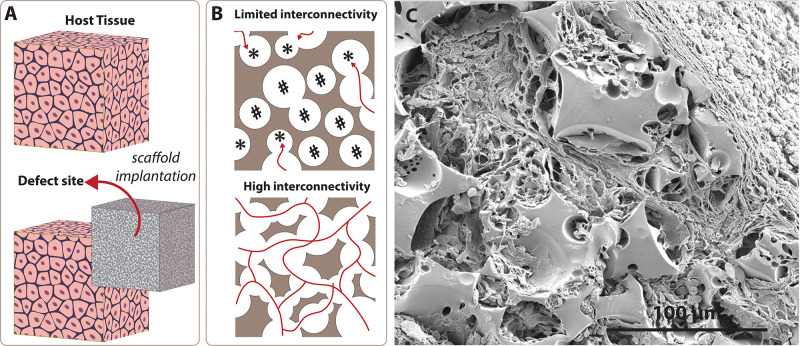
Significance of the interconnectivity on scaffold design. **(A)** Scaffolds are 3D substrates that are implanted to the defect site to guide tissue regeneration. **(B)** Low interconnectivity limits cell infiltration due to blind (labelled with *) and inaccessible (labelled with #) pores, while higher interconnectivity provides enhanced permeability and cell penetration. **(C)** Scanning electron microscope image of the emulsion templated scaffold (polycaprolactone PolyHIPE) that shows tissue infiltration through the interconnected pores of the scaffold.

Tissue and organ failure is one of the most frequent, inevitable major public health problems due to congenital health issues, traumas, diseases, and the increasing average age of the population (Langer and Vacanti, 1993; Dzobo et al., 2018). Tissue Engineering (TE) aims to devise solutions to restore or to improve the functions of injured/diseased parts of the host tissue which cannot heal naturally. TE utilises porous matrices that are called scaffolds to fill the defect site (Figure 1A). Scaffolds serve as a guide for tissue regeneration as a three-dimensional substrate for cell attachment, proliferation, infiltration, and they also provide temporary mechanical support. There are five essential requirements that an ideal scaffold should have (O’Brien, 2011; Bose et al., 2012); (i) biocompatibility, not causing any adverse effect at any level, from cellular activity to molecular signalling, on cells/tissues when they are in contact (Williams, 2008; Bose et al., 2012), (ii) biodegradability, degrading over time *in vivo* to create a space for newly forming tissues, (iii) having appropriate surface chemistry to allow cellular attachment, proliferation and differentiation, (iv) having similar mechanical properties with the native tissue not to fail tissue formation due to excessive deformation (Hollister, 2005; Bose et al., 2012), and (v) the morphology is the key feature that affects both biological and mechanical efficiency of the scaffolds. Scaffolds are needed to have a porous architecture with high interconnectivity to enable cell infiltration, nutrient flow, and integration of the material within the host tissue (Figure 1B).

To date, various scaffold manufacturing techniques such as gas foaming (Salerno et al., 2009; Bak et al., 2014), porogen leaching (Reignier and Huneault, 2006; Bak et al., 2014), electrospinning (Aldemir Dikici et al., 2019a; Mangir et al., 2019a; Dikici et al., 2020b,c), and additive manufacturing (AM) (Elomaa et al., 2011; Aldemir Dikici et al., 2017) have been widely used to introduce porosity into TE scaffolds. Recently, emulsion templating has gained particular attention as a scaffold fabrication technique due to its three main advantages; providing (i) high porosity[(up to 99%) (Richez et al., 2005)], (ii) high interconnectivity (Figure 1C), and (iii) high tunability. While high porosity and interconnectivity enable cell migration, vascularisation, and providing space for newly forming tissues (O’Brien, 2011; Loh and Choong, 2013), high tunability of physical, chemical and mechanical properties of emulsion templated matrices enables fabrication of precisely engineered scaffolds to meet the requirements of specific TE applications.

**FIGURE 2:**
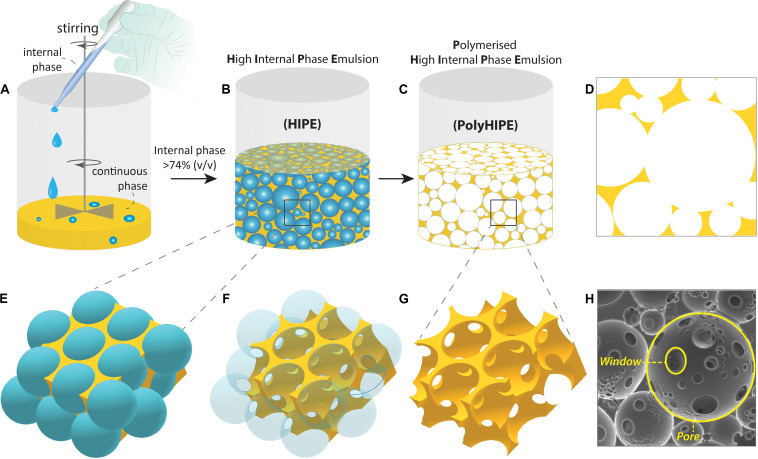
Fabrication steps of the Polymerised High Internal Phase Emulsion (PolyHIPE). **(A,B)** The gradual addition of the internal phase into the continuous phase while the system is mixed, **(C)** polymerisation of the high internal phase emulsion (HIPE), **(D)** 2D projection of PolyHIPE, **(E–G)** the formation of the pores and windows, and **(H)** scanning electron microscope image of the PolyHIPE.

The technique is based on two basic steps; the preparation of emulsion composed of at least two immiscible liquids where one phase (internal phase, dispersed phase) dispersed in the other phase (continuous phase, external phase) and solidification of the continuous phase of the emulsion. In this process, droplets of dispersed phase behave like templates, and they are removed following solidification to obtain porous matrices (Figure 2). These biphasic emulsion systems can be either water-in-oil (w/o) or oil-in-water (o/w) depending on the positioning of the lipophilic (non-polar, fat-loving, oil) and hydrophilic (polar, water-loving, water) phases.

**FIGURE 3:**
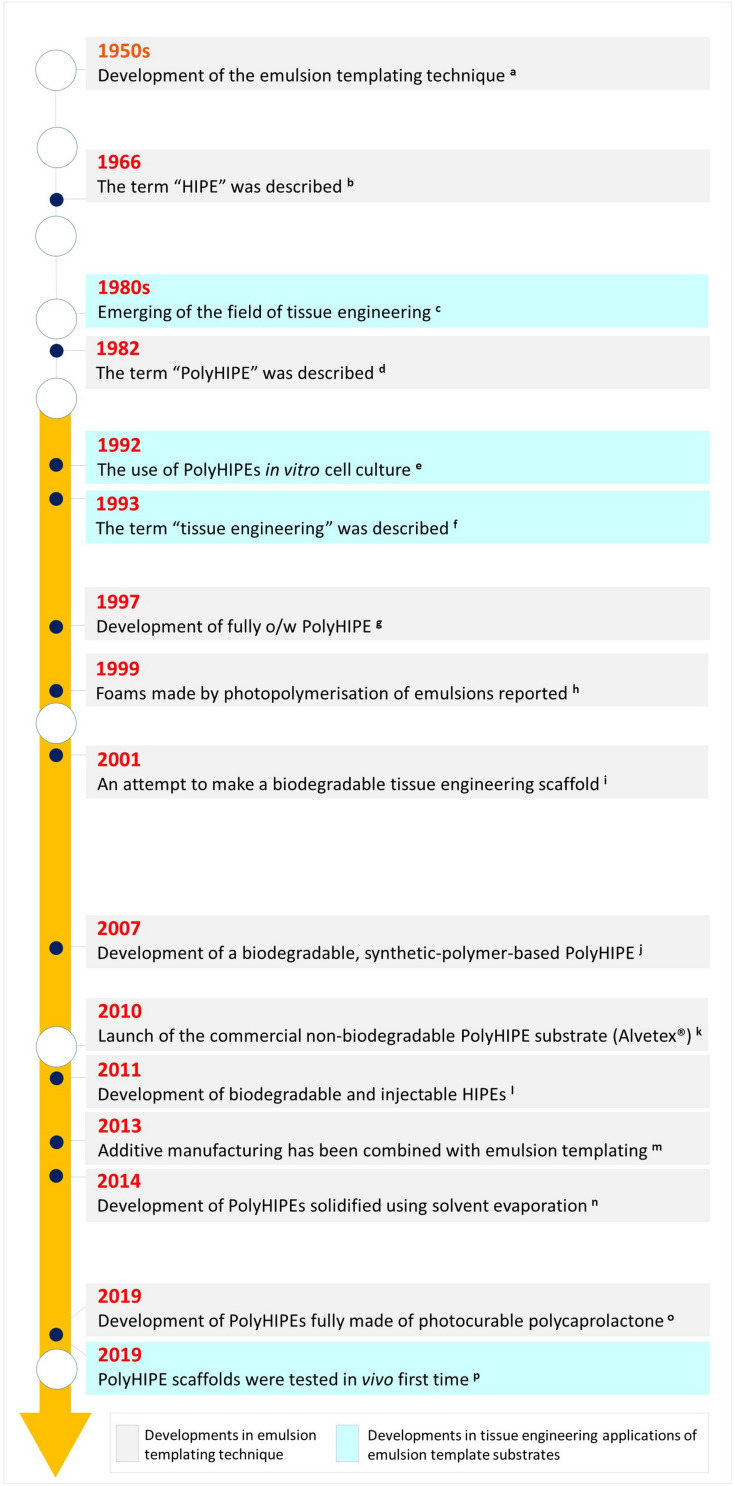
Historical landmarks in emulsion templating in terms of material development and its use in tissue engineering applications [a. (Guenther, 1959), b. (Lissant, 1966), c. (Vacanti, 2006; Meyer, 2009), d. (Barby and Haq, 1985), e. (Lee et al., 1992a,b), f. (Langer and Vacanti, 1993), g. (Kitagawa, 2001), h. (Thunhorst et al., 2003), i. (Busby et al., 2001), j. (Christenson et al., 2007), k. (Padbury, 2019), l. (Moglia et al., 2011), m. (Johnson et al., 2013; Sušec et al., 2013), n. (Hu et al., 2014a), o. (Aldemir Dikici et al., 2019b), p. (Aldemir Dikici et al., 2019a)].

Although emulsion templating has been mentioned as a relatively new scaffold manufacturing route in recent publications, the birth of the term of emulsion templating in the literature is older than TE itself (Figure 3); it dates back to the late 1950s (Guenther, 1959) where it was defined in a patent. Many other patents -including one by National Aeronautics and Space Administration (NASA)- followed up the development of emulsion templated polymers for different applications such as oil absorbents (Fletcher and Marsh, 1977) and 3D shaped porous objects with smooth surfaces (Guenther, 1971). Over the years, emulsion templated matrices have been used in various other areas such as; catalyst supports (Zhang Y. et al., 2016), separation columns (Yang et al., 2010), heavy metal removal (Mert et al., 2012), solid-phase synthesis (Small and Sherrington, 1989), and substrates for electrodes (Brown and Sotiropoulos, 2001).

**FIGURE 4:**
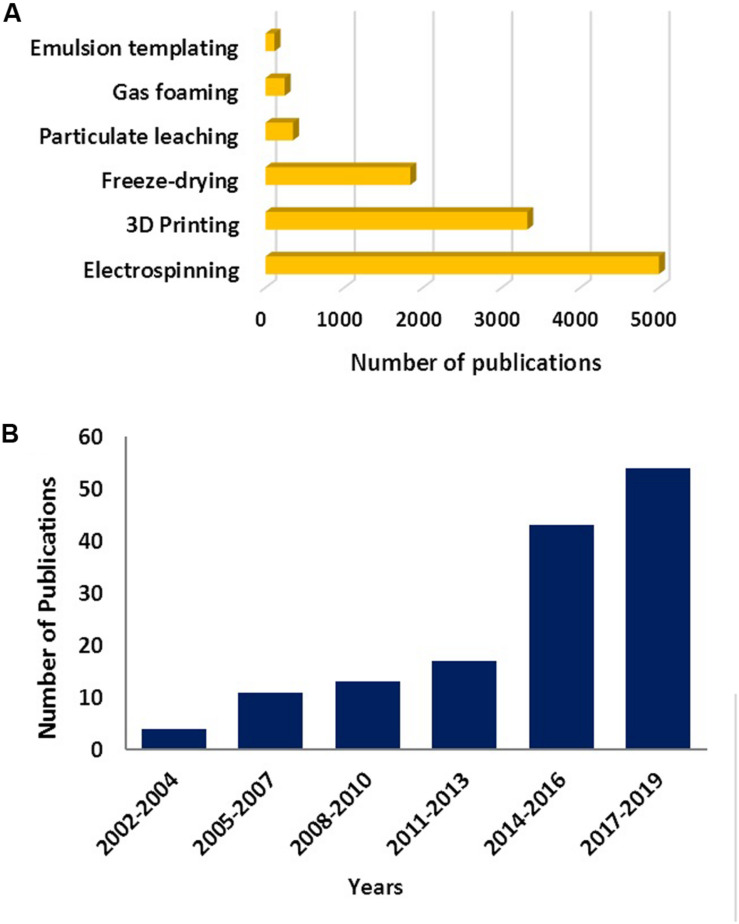
**(A)** The number of publications between 1900 and 2019 (Data obtained on 22 Nov 2019) for various scaffold fabrication methods. Data generated using –the name of the manufacturing route- and “tissue engineering” as a search term. **(B)** The number of papers published on emulsion templating in TE in the given years. Data generated using “emulsion templating” and “tissue engineering” as search terms. Web of Science was used as a search platform, and keywords were searched in all the fields.

Studies on the use of emulsion templating for the manufacturing of 3D substrates for cell culture applications is relatively new; it dates back to the early 1990s (Lee et al., 1992a,b; Schrimpf and Friedl, 1993; Akay et al., 2000). That is most likely why the number of TE-related studies is not more than 6% of the total reported number emulsion templating publications. Emulsion templating also has the lowest number of reports on TE applications when compared with other well-known scaffold manufacturing techniques (Figure 4A). However, there has been an increasing trend in the number of publications on emulsion templating in the last years, and almost 40% of emulsion templating in TE papers have been published in the last 3 years (Figure 4B).

Development of the emulsion templated scaffolds requires a multidisciplinary approach that combines knowledge and experience from chemistry, materials science, and TE. To date, there has been a number of significant reviews from the pioneers in the field of emulsion templating in the literature (Cameron, 2005; Silverstein and Cameron, 2010; Pulko and Krajnc, 2012; Silverstein, 2014a,b; Zhang et al., 2019). These reviews comprehensively cover the chemistry and material science behind this technique and briefly summarise all of the potential usage areas of emulsion templating. Accordingly, in this review, we aimed to approach emulsions templating as solely a TE scaffold fabrication technique. We summarised the basics of emulsion templating by reviewing the literature and determined a road map for the researchers that would like to explore this advantageous technique in their TE applications and reported the current state of the art of emulsion templating in TE as a retrospective.

## Polymerised High Internal Phase Emulsions: Terminology

**FIGURE 5:**
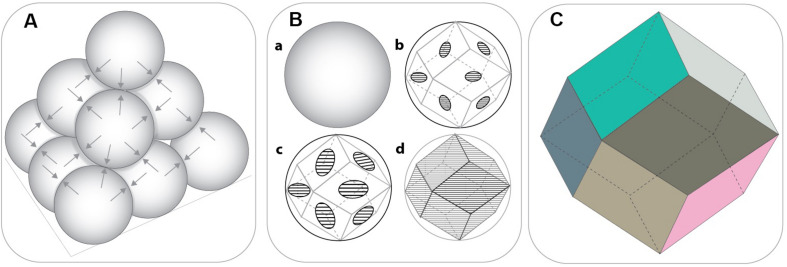
**(A)** Closest packing density of the solid spheres (non-deformed) (74.048%) where each sphere touches twelve other spheres. **(B)** The transition from sphere to rhomboidal dodecahedron (RDH) by the gradual flattening of the touching points. **(C)** The geometry of the basic RDH. [Adapted images were recreated using Ref. Lissant (1966) as a guide].

One of the most favourable features of emulsion templated scaffolds is the tunability of their porosity by simply increasing the internal phase volume. In the literature, emulsions that have at least 74.048% internal phase volume are defined as High Internal Phase Emulsions (HIPEs). The value of 0.74048 (π/√18) is the densest possible monodispersed sphere packing density, according to Kepler Conjecture (Hales, 2005). Also, according to Oswald’s phase volume theory, this value corresponds to monodispersed, undistorted, hexagonal-packed droplets (Princen, 1979). Beyond this value, emulsions have been assumed to tend to break unless the emulsion is heterodisperse, because the heterodispersity will enable smaller droplets to fill the intersects of the bigger droplets, and this results in higher internal phase volume for the emulsion. Lissant (1966) reported that it is possible to prepare relatively mono-disperse emulsions with internal phase beyond 74.048% by the right choice of surfactant and demonstrated that monodispersed water droplets deformed into polyhedrons as the touching points become flattened (Figure 5).

This situation applies in monodispersed spheres, and already in 1907 Spencer Pickering questioned the validity of this value for liquid spheres as liquid droplets in emulsion are in reality not uniform but are polydisperse (Pickering, 1907). Additionally, in emulsions, liquid droplets are not actually in contact; there is a thin wall separating the droplets from each other. That’s why, even the maximum packing density for mono-disperse, non-deformed liquid droplets would be less than the maximum packing density of the solid spheres (<74.048%). Indeed, the requirement for a revised definition of HIPEs has also been highlighted recently by other researchers (Menner et al., 2006; Manley et al., 2009; Aldemir Dikici et al., 2019b). However, herein, we use the commonly recognised definition of HIPEs.

Although emulsion polymerisation has previously been described in the literature (Bartl and Von Bonin, 1962; Bartl and von Bonin, 1963), the term “Polymerised High Internal Phase Emulsion (PolyHIPE)” appeared in the literature in 1982 to define porous structures formed following solidification of the HIPEs (Barby and Haq, 1985). Emulsion templated matrices with various internal phase volumes; PolyHIPEs [∼74–99% (Robinson et al., 2014)], Polymerised Medium Internal Phase Emulsions (PolyMIPEs) (30–74%) and Polymerised Low Internal Phase Emulsions (PolyLIPEs) [<30% (Zhang et al., 2019)] have been reported in the literature. However, in this review, we mainly focus on PolyHIPEs unless otherwise stated.

In terminology, it is important to comprehend the difference between HIPEs and PolyHIPEs. HIPEs can be further processed until the gelation point to change their droplet size and viscosity, but PolyHIPEs are the solid matrices that are made of solely the continuous phase, and they are obtained by the polymerisation of HIPEs. The cavities formed after removal of the internal phase being defined as “pores,” “cells,” or “voids.” The throats connect the adjacent pores to each other are defined as “interconnects” or “windows” (Figure 2). The formation of these interconnects has been reported to be due to the rupture of the thin polymer films between neighbouring pores during the polymerisation (Cameron et al., 1996). Depending on the presence and absence of interconnects, PolyHIPEs are categorised as “open cellular” and “closed cellular,” respectively.

## Characteristics of HIPEs and PolyHIPEs

Emulsion templating combines two main research areas that are emulsion science and porous polymer fabrication. Emulsions have been widely used for various industries, such as food, petroleum, paint, pharmaceutical, and cosmetic, for centuries (Grace, 1992; Fox et al., 2012). Also, porous polymer matrices/polymer foams have been fabricated by following a great variety of other routes (Jin et al., 2019). Thus, the characteristics of emulsions such as droplet size and viscosities (Goodarzi and Zendehboudi, 2019) and the characteristics of polymer foams such as morphological, mechanical features (Ceglia et al., 2012; Jin et al., 2019) are well-reported in the literature, and these characteristics are also mostly valid for HIPEs and PolyHIPEs, respectively. However, in this section, it is crucial to summarise these characteristics briefly to introduce the parameters that can be controlled and precisely engineered for specific applications by simply changing their composition and process conditions which are detailed in section “Development of the Emulsion Templated Scaffolds.”

### Morphological Characteristics

Internal phase volume is the main factor determining the porosity of emulsion templated scaffolds. However, the volume of the internal phase does not always correspond to the porosity of PolyHIPE scaffolds. In our recent study, the porosity of polycaprolactone (PCL) PolyHIPEs prepared using 82% internal phase volume was measured around 70%. This is likely because of 15–20% shrinkage of these scaffolds in each dimension during crosslinking and drying (Aldemir Dikici et al., 2019b). This is corroborated by other studies, for example, Chen et al. (2018) also reported a lesser extent of porosity than the internal phase volume of poly(styrene-co-2-ethylhexylacrylate) PolyHIPEs due to the same reason. They also reported that the extent of the shrinkage depends on the fractions of the 2-ethyl hexyl acrylate (EHA) and styrene in the composition of the PolyHIPE.

Thus, the porosities of the PolyHIPEs can be calculated using Eq. 1 (Ovadia and Silverstein, 2016; Barbara et al., 2017; Aldemir Dikici et al., 2019b), where ρ_PolyHIPE_ is the PolyHIPE density and ρ_wall_ is the density of PolyHIPE wall. The measured density of the bulk polymer is used for the density of the wall.

(1) $$\%\mathrm{Porosity}=\left(1-\frac{\rho_{\text{PolyHIPE}}}{\rho_{\text{wall}}}\right)\times 100$$

It has been reported that emulsion templated scaffolds may shrink in a dry state depending on their chemical compositions (Pierre et al., 2006; Murphy et al., 2017). On the contrary, they may swell in various solvents to a certain extent. For example, PEG diacrylate (PEGDA) PolyHIPEs has been reported to have swelling ratio up to 700% as a whole scaffold (material and pores), and 60% as a scaffold material (not accounting pores) (Murphy et al., 2017) in PBS and this swelling process may cause expansion of the pores (Ovadia and Silverstein, 2016). Thus, pore sizes and the porosities of those may be different at their wet and dry conditions. However, for TE scaffolds, we assume that the pore size and porosity in the wet state (in a physiologically relevant aqueous solution) is more relevant as the scaffolds are introduced into a fluid-rich environment in the body.

**FIGURE 6:**
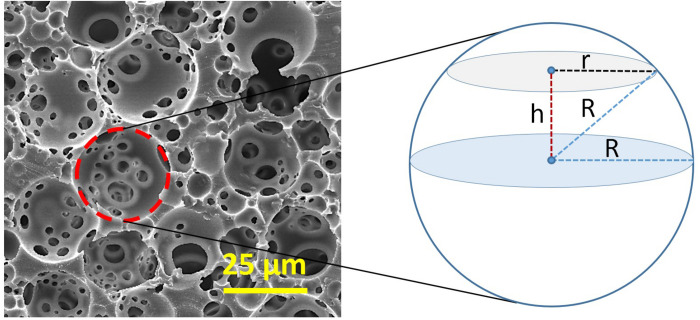
Derivation of the statistical correction factor that is applied for adjusting the underestimation of the exact diameter of the pore size. *R*, an actual radius of the pore and r, the radius of the circular section at a distance of h from the centre of the pore.

Higher volume of the internal phase causes a reduction in the pore size as tighter packing of the droplets is needed (Sušec et al., 2015). Typically, the average pore size and window size ranges of PolyHIPEs are 1-150 μm and 0.2–50 μm, respectively (Barbetta et al., 2005b; Robinson et al., 2014). Mercury porosimetry and nitrogen adsorption methods are effective tools to characterise the structural and physical features of PolyHIPEs (Barbetta and Cameron, 2004). Another commonly used method is measuring the pore and window sizes using scanning electron microscopy (SEM) images of the cross-section of the PolyHIPEs. However, when the scaffolds are sectioned, pores are not ideally bisected; they are sectioned from a random distance (*h*) from the centre (Figure 6). Thus, as we can only measure the radius of the circular section at a distance of h from the centre of the pore (*r*) rather than the exact pore radius (*R*), a statistical correction factor should be applied to the measured average pore size (Barbetta and Cameron, 2004). The relationship between *R*, *r*, and *h* can be expressed using Eq. 2.

(2) $$R^{2}=h^{2}+r^{2}$$

The value of *h* can be between 0 to *R*, depending on the position of the sectioning. By replacing an average value for *h*; *R*/2, in Eq. 2, *R*/*r* can be found 2/√3 as a correction factor that is applied to the measured diameter for adjusting the underestimation of the exact diameter.

The degree of interconnectivity of PolyHIPEs is calculated by dividing the average window size to average pore size (*d*/*D*) (Carnachan et al., 2006; Aldemir Dikici et al., 2019b) however, as this number does not give any indication about the number of windows, an alternative definition of the degree of openness, which is suggested to be calculated by dividing open surface area of the pore to total surface area of the pore (Eq. 3) (Pulko and Krajnc, 2012; Owen et al., 2016).

(3) $$\mathrm{Degree}\,\mathrm{of}\,\mathrm{openness}=\frac{\begin{array}{c} \mathrm{Open}\,\mathrm{surface}\,\mathrm{area}\,\mathrm{of}\,\mathrm{the}\,\mathrm{pore}\\ \left(\mathrm{area}\,\mathrm{of}\,\mathrm{the}\,\mathrm{windows}\right) \end{array}}{\mathrm{Total}\,\mathrm{surface}\,\mathrm{area}\,\mathrm{of}\,\mathrm{the}\,\mathrm{pore}}$$

### Physical Characteristics

PolyHIPEs are defined as low-density polymeric foams with typical densities of around 0.1 g/cm^3^ (Silverstein et al., 2011). This value can be lowered with higher porosity and a higher degree of openness, and it has a direct effect on the mechanical properties of the matrices (Kravchenko et al., 2018). PolyHIPEs are also characterised with the low surface area due to the openness on the cavities. While increasing internal phase volume reduces the surface area dramatically (Cameron, 2005), the addition of porogenic solvents can increase the surface area up to 690 m^2^/g. PolyHIPEs with significantly higher surface area (up to 2,000 m^2^/g) can be obtained using the hyper-crosslinking approach (Pulko et al., 2010; Mezhoud et al., 2018).

### Rheological Characteristics

HIPEs are viscous emulsions, and they have a mayonnaise-like consistency with yellowish-white colour due to the difference in light refraction between the oil and water phases. They exhibit shear-thinning behaviour (Sears et al., 2016; Bhagavathi Kandy et al., 2018; Aldemir Dikici et al., 2020). The viscosity of HIPEs depends on the viscosities of the internal and continuous phases, droplet size, and the internal phase volume (Ford and Furmidge, 1967; Das et al., 1992; Borwankar and Case, 1997; Welch et al., 2006; Ilia Anisa and Nour, 2010).

### Mechanical Characteristics

The mechanical features of PolyHIPEs can be tailored to a large extent by tuning their composition (Caldwell et al., 2012; Owen et al., 2016) and morphology (Karageorgiou and Kaplan, 2005; Huš and Krajnc, 2014; Owen et al., 2015, 2016; Aldemir Dikici et al., 2019b; Kovačič et al., 2019). Although high porosity is desired for better cell infiltration in TE scaffolds, there is an indirect relationship between porosity and mechanical properties of porous foams (Karageorgiou and Kaplan, 2005; Owen et al., 2015, 2016). Similarly, the higher degree of interconnectivity results in a larger open area within the walls, and it leads to lower structural integrity (Barbetta and Cameron, 2004; Aldemir Dikici et al., 2019b).

The Young’s modulus of the porous foams has been shown to increase by increasing pore size and relative density (Jiang et al., 2007; Lin-Gibson et al., 2007; Huš and Krajnc, 2014; Aldemir Dikici et al., 2019b; Kovačič et al., 2019). Also, the impact of strut thickness, shape, and other morphological parameters on the mechanical properties of porous materials has been shown using theoretical models and experimental studies (Li et al., 2006; Gholami et al., 2017).

## Development of the Emulsion Templated Scaffolds

**FIGURE 7:**
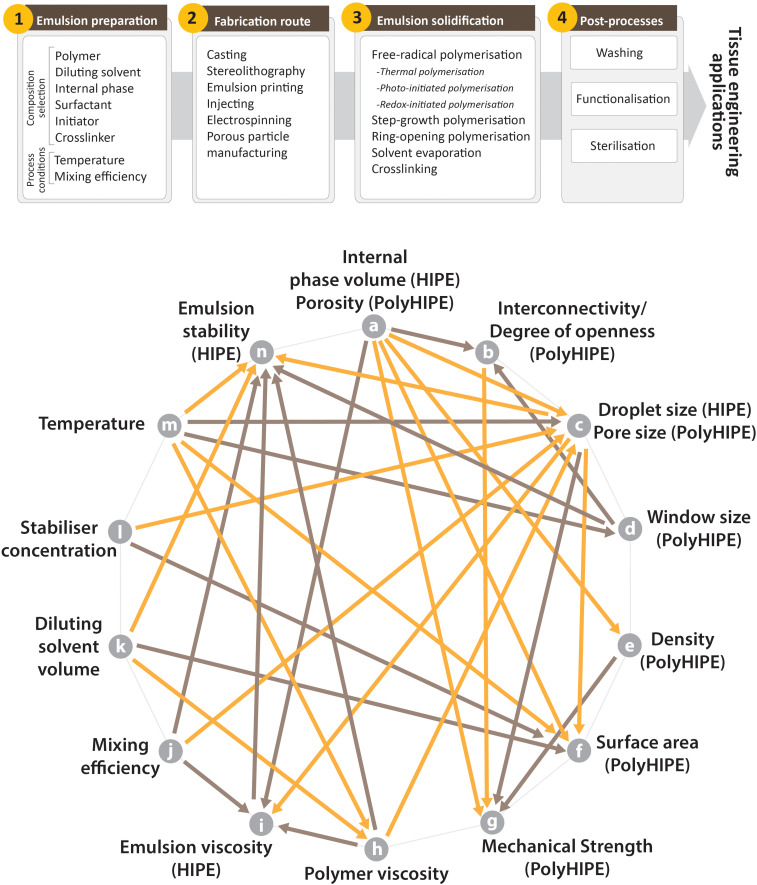
**(Top)** Steps of manufacturing of the emulsion templated substrates, **(Bottom)** commonly seen relations of the characteristics of HIPEs and PolyHIPEs and some of the process conditions (orange arrows indicate one-way reciprocal proportionality, brown arrows indicate one-way direct proportionality. For example, an increase in the porosity of PolyHIPEs (or internal phase volume of HIPEs – characteristic of HIPEs and PolyHIPEs corresponds to each other) reduces the density of PolyHIPEs and increases the emulsion viscosity. References for the relations; (a–i) (Pal and Rhodes, 1985; Cameron and Sherrington, 1996; Farah et al., 2005), (a–g) (Karageorgiou and Kaplan, 2005; Owen et al., 2015, 2016), (a–e) (Kravchenko et al., 2018), (a–c) (Sušec et al., 2015; Kravchenko et al., 2018), (a–b) (Barbetta et al., 2005b; Owen et al., 2016), (a–f) (Cameron, 2005), (b–g) (Barbetta and Cameron, 2004; Owen et al., 2016; Aldemir Dikici et al., 2019b), (c–i) (Zhou et al., 2019), (c–g) (Lin-Gibson et al., 2007; Huš and Krajnc, 2014; Aldemir Dikici et al., 2019b; Kovačič et al., 2019), (c–f) (Cameron, 2005), (c–n) (Lim et al., 2015), (d–b) (Pulko and Krajnc, 2012), (d–n) (Cameron, 2005), (e–g) (Kravchenko et al., 2018), (h–c) (Aldemir Dikici et al., 2019b), (h–n) (Aldemir Dikici et al., 2019b), (h–i) (Barnes, 1994), (i–n) (Lim et al., 2015), (j–i) (Fan et al., 2018; Zhou et al., 2019), (j–c) (Moglia et al., 2011; Paterson et al., 2018; Zhou et al., 2019), (j–n) (Fan et al., 2018), (k–h) (Aldemir Dikici et al., 2019b), (k–f) (Cameron, 2005; Pulko et al., 2010), (k–n) (Carnachan et al., 2006; Aldemir Dikici et al., 2019b), (l–c) (Moglia et al., 2011), (l–f) (Wang et al., 2020), (m–h) (Aldemir Dikici et al., 2019b), (m–n) (Carnachan et al., 2006; Lim et al., 2015), (m–c) (Carnachan et al., 2006; Caldwell et al., 2012; Paterson et al., 2018), (m–d) (Carnachan et al., 2006), (m–f) (Huš et al., 2015).

Emulsion templated scaffolds are fabricated by following a multistep route before they are used in TE applications (Figure 7); (i) development of the emulsions by optimisation of their composition and emulsification conditions, (ii) structuring the emulsions, (iii) applying the appropriate solidification method, and (iv) post-processing which include improving the functionality of the scaffolds, purification, and sterilisation.

### Preparation of HIPEs

There are at least three essential ingredients that need to be used to make HIPEs; (i) a continuous phase (polymer phase), (ii) an internal phase, and (iii) a stabiliser [although there is a limited number of studies on the development of stabiliser-free HIPEs (Oh et al., 2015)]. In addition to these core elements of the HIPEs, additional ingredients may be required to be added into the inner or/and into the continuous phase of the emulsion.

#### Formulation of the Continuous Phase

The selection of the monomeric or oligomeric pre-polymer for the continuous phase of the PolyHIPEs is the fundamental basis to formulating emulsion templated structures with pre-determined properties and will be discussed in section “Monomers/Macromers.” The pre-polymer is typically formulated with a number of additives (i.e., solvent, stabiliser and initiator) to form the continuous phase, which will be discussed in this section.

#### Diluting Solvents

Pre-polymers used in the emulsification process may be in solid-state or in liquid phase with high viscosity. During the mixing of the two immiscible phases, although the high viscosity of the continuous phase increases the kinetic stability of the emulsion, it needs to be low enough to enable efficient mixing of the two phases (Kuhlmann, 2000; Christenson et al., 2007). In order to reduce the viscosity of the polymer phase, either the temperature of the system can be increased (Figure 7), or polymers can be diluted with the solvents that are called diluting or porogenic solvents as they are removed after polymerisation. After removal, these matrices shrink up to 50% (Busby et al., 2002). Also, the addition of diluting solvents may provide additional nanoscale porosity on the walls of the PolyHIPEs (Silverstein et al., 2005).

Diluent type (Aldemir Dikici et al., 2019b) and volume (Christenson et al., 2007; Aldemir Dikici et al., 2019b) plays a critical role in the characteristics of HIPEs and PolyHIPEs. While water and phosphate buffer saline (PBS) are commonly used to dilute the continuous phase of the o/w emulsions (Oh et al., 2015), more apolar solvents (with less solubility in water) such as; toluene (Busby et al., 2001; Christenson et al., 2007; Changotade et al., 2015; Aldemir Dikici et al., 2019b), chloroform (Aldemir Dikici et al., 2019b), tetrahydrofuran (THF) (David and Silverstein, 2009), dichloromethane (DCM), and dichloroethane (DCE) (Johnson et al., 2015) are used as diluents in w/o emulsions.

Recently, we have shown the impact of absence/presence, volume and the type of diluting solvents on the stability of PCL HIPEs and the morphology of PCL PolyHIPEs (Aldemir Dikici et al., 2019b). Increasing the volume of the diluent enhances the limit of the maximum internal phase volume that can be incorporated into the emulsion. However, a further increase in the solvent volume from a certain point reduces stability HIPE (Aldemir Dikici et al., 2019b). Thus, there is a narrow range that a stable emulsion can be formed. The viscosity should be low enough to enable mixing of the two phases, but high enough to form a stable emulsion.

#### Internal Phase (Dispersed Phase)

While the internal phase of w/o emulsions is most dominantly composed of water, in reversed emulsions (o/w), more apolar liquids, often toluene (Barbetta et al., 2005a,b; Krajnc et al., 2005) form the internal phase. Selection of the internal phase composition and the volume is another factor that has an impact on the properties of HIPEs and PolyHIPEs. Krajnc et al. (2005) tested toluene, chlorobenzene, DCM, and chloroform as the internal phases for acrylic acid PolyHIPEs (o/w), and reported that only the emulsions prepared with toluene resulted in as a stable emulsion.

There are some salts such as sodium sulphate (Na_2_SO_4_), calcium chloride (CaCl_2_), sodium chloride (NaCl) (Pons et al., 2007), and potassium iodide (KI) that are included in the internal phase of the w/o emulsions to increase stability (Kunieda et al., 1989; Rajagopalan et al., 1994). Opawale et al. showed that NaCl affects the surfactant adsorption and the emulsions interfacial elasticity, which play a crucial role in emulsion stability (Powell et al., 2017). However, the intensity of the impact depends on the type of surfactant used (Opawale and Burgess, 1998). CaCl_2_ was reported to increase emulsion stability by preventing Oswald ripening (Moglia et al., 2011). Similarly, potassium sulphate (K_2_SO_4_) was also reported to increase the rigidity of the interface between the two phases (Lumelsky et al., 2008, 2009; Lumelsky and Silverstein, 2009).

Opposed to abovementioned applications that attempt to increase the stability of the emulsions, there are some approaches to reduce emulsion stability on purpose to enable an increase of the pore size of the PolyHIPE. Magnesium sulphate (MgSO_4_) has been reported to reduce the surfactant adsorption and increases the droplet size within the emulsion (Kent and Saunders, 2001). Also, chemicals that are partially soluble in both phases of the emulsion such as poly(ethylene glycol) (PEG) (Carnachan et al., 2006) and THF (Hayman et al., 2004, 2005; Cameron, 2005; Carnachan et al., 2006) reduce emulsion stability and are included in the internal phase of the w/o emulsions to increase pore size.

#### Stabilisers

The coexistence of two immiscible liquids in the emulsion composition causes high surface tension at the interfaces of these liquids. The droplets of the inner phase coalescence gradually to reduce the surface area, and this process ends up inevitably as phase separation. According to surface tension theory, stabilising agents reduce the interfacial tension by stabilising the oil-water interface (Khan et al., 2011; Kale and Deore, 2016).

##### Surfactant stabilisation

**FIGURE 8:**
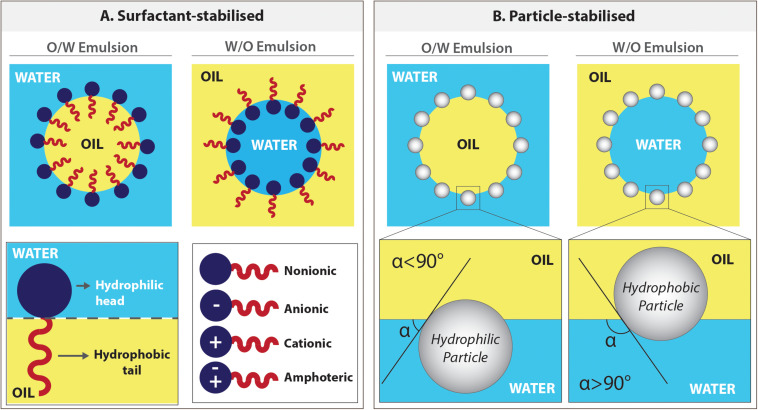
Emulsions can be stabilised either by surfactants or particles. **(A)** The positioning of the surfactant molecules in o/w and w/o emulsions and types of surfactants. **(B)** The positioning of the particles on the water–oil interface of either in o/w or w/o emulsions depending on the degree of wettability of the particles by these phases.

The surfactant is an amphiphilic compound; that its head is water-soluble, and the tail is oil-soluble (Figure 8). Surfactants create a continuous film around the inner phase, act as a barrier between two phases which reduces the interfacial tension, and stabilises the emulsion. There are various types of surfactants available, and they are classified as non-ionic, anionic, cationic, and amphoteric, depending on the charge of the hydrophilic head (Figure 8A). The surfactant choice (Busby et al., 2002; Moglia et al., 2011) and concentration (Aldemir Dikici et al., 2019b) play an important role in emulsion stability and PolyHIPE morphology.

When there is no surfactant used in the water and oil system, the type of emulsion depends on the volume of the phase. The higher volume phase will mostly be the continuous phase. In the presence of surfactants, according to the Bancroft Rule (Bancroft, 1913), the phase that the surfactant is predominantly dissolved in forms the continuous phase. Specifically, while oil-soluble surfactants tend to form w/o emulsions, water-soluble surfactants are suitable for o/w emulsions (Finkle et al., 1923).

Although the selection of the best working surfactant has been empirical, the quantitative classification of the surfactants, the hydrophile-lipophile balance (HLB) classification described by Griffing, gives an insight for the initial surfactant choice (Griffin, 1946, 1954). The HLB value varies between 0 to 20, and the value is in direct correlation with the hydrophilicity of the surfactant (Myers, 2005). While surfactants with low HLB values are good for w/o emulsions, surfactants with high HLB values are more suitable for o/w emulsions (Myers, 2005).

The HLB value of a surfactant is not always a sole determining factor for emulsion stability, which depends on various parameters such as the selection of the monomer and solvent, emulsification temperature, and absence/presence of the electrolyte (Myers, 2005). Indeed, many researchers reported that just HLB is not enough on its own to select a suitable surfactant for emulsion systems (Moglia et al., 2011).

W/o emulsions are mostly stabilised using oil-soluble non-ionic surfactants (Silverstein, 2014b). Span 80, Hypermer 246 and polyglycerol polyricinoleate (PGPR) are the most widely used surfactants for w/o HIPEs (Table 1). However, Zhang et al. replaced the non-ionic surfactant with cationic surfactant for divinylbenzene (DVB)-styrene HIPEs and reported the formation of PolyHIPEs with higher pore volumes than the ones prepared using non-ionic surfactants (Zhang and Chen, 2007). The concentration of the surfactants used in the preparation of HIPEs is generally in the range of 1–30% (w/w) (of the monomer). A higher surfactant concentration results in smaller average pore size and more uniform pore size distribution (Moglia et al., 2011).

**TABLE 1:** Commonly used surfactants in HIPEs of various polymer systems.

**Emulsion type**	**Emulsifier**	**HLB/type**	**Polymer**	**References**
w/o	Polyglycerol polyricinoleate (PGPR)	∼3 (Surh et al., 2007) Nonionic	1,6-Diisocyanatohexane and Polycaprolactone triol	David and Silverstein, 2009
			Propylene fumarate dimethacrylate	Moglia et al., 2011, 2014c; Robinson et al., 2014
	Sorbitan monooleate (Span 80)	4.3 (Kassem et al., 2019) Nonionic	Propylene fumarate (diacrylate)	Christenson et al., 2007
			Styrene/divinylbenzene	Akay et al., 2004; Hayman et al., 2005
			2-Ethylhexyl acrylate (EHA) and isobornyl acrylate (IBOA)	Pierre et al., 2006
	Hypermer 246	5–6 (Audouin and Heise, 2014) Nonionic	2-Ethylhexyl acrylate (EHA) and isobornyl acrylate (IBOA)	Johnson et al., 2013; Malayeri et al., 2016; Owen et al., 2016; Wang et al., 2016
			Thiolene	Lovelady et al., 2011; Richardson et al., 2019
			Polycaprolactone tetramethacrylate	Aldemir Dikici et al., 2019a,b
	Brij-58	15.7 (Thakare et al., 2017) Nonionic	Polycaprolactone	Samanta et al., 2016a
	Pluronic L121	1 (Amiji, 2004) Nonionic	Tetrakis-3-mercaptopropionate and divinyladipate	Naranda et al., 2016
	Cetyltrimethylammonium bromide (CTAB)	10 (Prakash, 2010) Cationic	Styrene/divinylbenzene	Zhang and Chen, 2007

o/w	Triton X-405	17.9 (Slinde and Flatmark, 1976) Nonionic	Alginate methacrylate	Barbetta et al., 2009; Zhou et al., 2013
			Dextran Dextran-b-PolyNIPAAm (Poly(N-isopropylacrylamide))	Zhou et al., 2012b
			Gelatin methacrylate	Barbetta et al., 2005a,b
			Pullulan methacrylate	Barbetta et al., 2005a
			Dextran methacrylate	Barbetta et al., 2005a
			Alginate	Barbetta et al., 2009
			Acrylic acid	Krajnc et al., 2005

Surfactants are commonly intended not to react with the monomer, and they are removed from the PolyHIPE composition following polymerisation. However, the use of reactive block copolymer surfactants in HIPE composition is also reported (Zhang and Silverstein, 2018). As they covalently attach to the PolyHIPE surface, surfactant removal is not needed.

##### Pickering particle stabilisation

The emulsion also can be stabilised using solid particles (micro or nanoparticles), and these surfactant-free emulsions are defined as Pickering emulsions (Gurevitch and Silverstein, 2010; Yang et al., 2017). As in surfactant stabilised emulsions, the pore size of the particle stabilised emulsions can be adjusted by changing the particle concentration (Zhou et al., 2012a).

The principle behind the stabilisation mechanism of Pickering emulsions is based on the wettability of the particles by oil and the water phases (preferential wetting theory) (Chang, 2016). These particles position on the interface and need to be absorbed by both phases to some extent. The particles are more adsorbed in the phase that they are wetted more, and this defines their positioning in the interface (Figure 8B). While water wetted particles forms o/w emulsions, oil-wetted particles can stabilise w/o emulsions (Schulman and Leja, 1954; Binks and Lumsdon, 1999; Pal, 2018). The particles that are not wetted by one of these phases disperse in the phase they are wetted and fail to stabilise the emulsion.

Hydroxyapatite (HA) is one of the most widely used particles for stabilisation of Pickering HIPEs. Interestingly it is reported to be used both o/w (Zhou et al., 2012a) and w/o (Hu et al., 2014a,b, 2015) HIPEs. Hu et al. (2019) used nano-HA to stabilise PCL HIPEs and in their follow-up study, Yang et al. claimed that emulsions stabilised using silica nanoparticles have a higher viscosity than the emulsions stabilised by HA particles (Yang et al., 2017). Starch nanoparticles (Kavousi and Nikfarjam, 2019) and gelatin nanoparticles (Tan et al., 2017) are the other alternative particles used for the stabilisation of the Pickering o/w and w/o emulsions, respectively.

#### Initiators

Initiators are chemical compounds that react with the monomers. They form intermediate compounds that can be linked with other monomers and propagate to form the polymer chains. Initiators can be included either into the inner or continuous phase of the emulsions, and the locus of initiation has been shown to have a significant effect on porous structures (Gurevitch and Silverstein, 2010).

Ammonium persulphate (APS) (Moglia et al., 2011) and potassium persulphate (KPS) (Lumelsky et al., 2008, 2009; Tunc et al., 2012) are water-soluble oxidising agents that are used as redox initiators in radical crosslinking of the macromer chains. They have been reported to be introduced into the water phase of the styrene (Bokhari et al., 2005; Audouin et al., 2012), dextran, (Zhou et al., 2012a) or polypropylene fumarate (PPF) (Christenson et al., 2007; Moglia et al., 2011) HIPEs with the concentration of 1–5% w/v of the aqueous phase.

Benzoyl peroxide (BPO) is an oil-soluble redox initiator also used for polymerisation of PPF HIPEs. Robinson et al. (2014) showed that initiator selection has a great impact on PolyHIPE morphology. While PPF PolyHIPEs prepared using APS as an initiator resulted in closed cellular structure, BPO included PolyHIPEs exhibited open cellular structure. Also, the concentration of the redox initiator has shown to have an impact on the curing time of HIPEs and on the mechanical properties of PolyHIPEs (Moglia et al., 2014c). 2,2′-Azobisisobutyronitrile (AIBN) is another oil-soluble initiator. It has been reported to be introduced into the continuous phase of styrene (Hayward et al., 2013a) and the internal phase of gelatin HIPEs (Barbetta et al., 2005b) for thermal polymerisation.

Photoinitiators are the molecules that create reactive species when exposed to light, and they are included in the composition of the HIPEs that will be polymerised via photo-initiation. In the photoinitiator selection process, the critical parameter is that the absorption band of the photoinitiator should overlap with the emission spectrum of the light source (Eibel et al., 2018). 2,4,6-Trimethylbenzoyl phosphine oxide/2-hydroxy-2-methyl propiophenone blend (Johnson et al., 2015; Sherborne et al., 2018; Aldemir Dikici et al., 2019a,b) and phenyl bis(2,4,6−trimethyl benzoyl)−phosphine oxide (BAPO) (Sears et al., 2016) are widely used photoinitiators in photo-polymerisation of HIPEs. Photoinitiators are mostly used in the HIPE composition at a concentration range of 0.2–10% (w/w) of the polymer. The concentration of the photoinitiator that is included in the composition of the photocurable resins is reported to have an effect on the rheological properties of the monomer and its gelation time (Mishbak et al., 2019). Interestingly, there are few studies that investigate the photoinitiator type and concentration on the characteristics of PolyHIPEs (Harikrishna et al., 2014).

#### Crosslinker (Crosslinking Agent)

**FIGURE 9:**
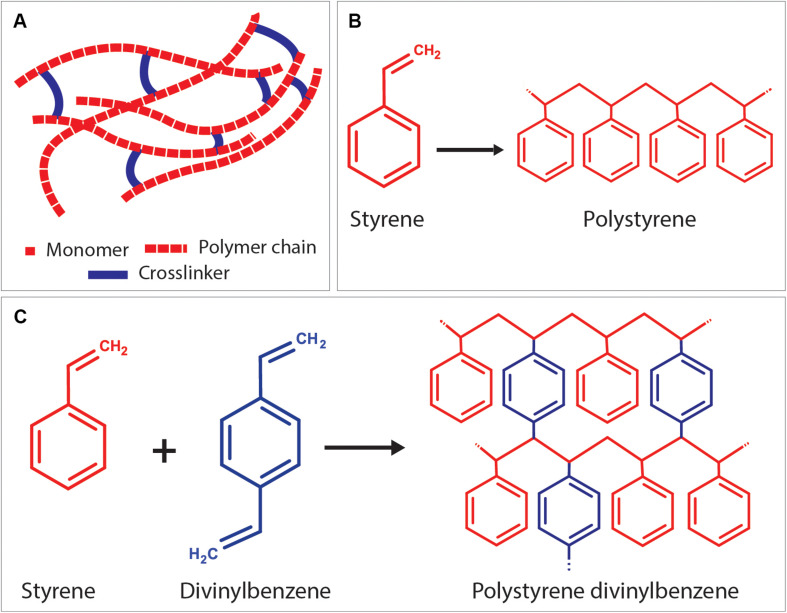
**(A)** Positioning of the crosslinker between linear polymer chains. As an example; **(B)** formation of polystyrene chain from styrene monomers, and **(C)** crosslinking of polystyrene chains using the crosslinker, divinylbenzene.

Crosslinkers are the precursors with at least two reactive ends to connect primary polymer chains by forming intermolecular linkages (Figure 9A). Using an external crosslinking agent increases the degree of crosslinking of the polymer phase, and it improves the stiffness of the materials (Tunc et al., 2012). The most known crosslinker is DVB, used in the composition of styrene HIPEs (Akay et al., 2004; Sevšek et al., 2014; Woodward et al., 2017; Figures 9B,C). Christenson et al. (2007) used propylene fumarate diacrylate (PFDA) as a crosslinker for PPF HIPEs and showed that crosslinker concentration has an impact on the emulsion stability of HIPEs and on the morphology of PolyHIPEs. Nalawade et al. verified this finding on hydrogel-based HIPEs (Nalawade et al., 2016). Trimethylolpropane triacrylate (TMPTA) is also widely used crosslinker for EHA and isobornyl acrylate (IBOA) PolyHIPEs (Malayeri et al., 2016; Owen et al., 2016).

#### Temperature

The most dramatic effect of the temperature is on the viscosity of the oil phase which also affects the viscosity and the stability of the emulsion. The viscosity of the polymer and the temperature are inversely related to each other as shown in Eq. 4 where *n* is the viscosity of the polymer, *T* is the temperature, *A* and *B* are material constants;

(4) $$n=A\,e^{\frac{B}{T}}$$

Also, according to Stoke’s equation (Eq. 5), the viscosity of the polymer and the velocity of the droplet (*v*) are inversely proportional (Murakami et al., 2014);

(5) $$v=D^{2}\,\Delta \,\rho \,g/18\,n$$

*D* is the droplet diameter under gravitational force, Δρ is the density difference between water and oil phase, *n* is the viscosity of the oil phase, and *g* is the gravitational force. Consequently, the increasing temperature reduces the oil phase viscosity, and this increases the speed of droplets of the inner phase and creates a bigger pore size (Bokhari et al., 2007b; Sušec et al., 2015; Paterson et al., 2018). Further increase in the temperature can lead to emulsion separation due to the increased mobility of the droplets (Carnachan et al., 2006; Caldwell et al., 2012). Although some studies increase the temperature in a controlled manner to increase the pore size, moderate temperatures are more favourable as they create comparatively more stable emulsions.

To investigate the effect of higher temperature on PolyHIPEs, researchers use different setups. Caldwell et al. showed that increasing the temperature of the inner phase from 23 to 80°C increased the pore sizes up to twofold (Caldwell et al., 2012). Akay et al. (2004) heated the whole mixing system using a stainless steel vessel with a heating jacket.

#### Efficiency of Mixing

In the conventional emulsification route, the internal phase is introduced into the continuous phase dropwise while the system is mixed continuously. There are various mixing methods reported; such as over-head stirrer (Lovelady et al., 2011; Caldwell et al., 2012; Johnson et al., 2015), magnetic stirrer (Aldemir Dikici et al., 2019a,b), mechanical shaking (Yang et al., 2017), speed mixer (Moglia et al., 2011, 2014c; Robinson et al., 2014), vortex (Richez et al., 2005), homogeniser (Hu et al., 2014b), and shaking by hand (Hu et al., 2015). The type of mixing is reported to have an effect on the maximum internal phase volume that could be incorporated into the emulsion (Richez et al., 2005). The effect of stirring speed on the characteristics of HIPEs has been reported by many groups (Moglia et al., 2011; Sušec et al., 2015; Paterson et al., 2018). Higher mixing speeds commonly result in smaller pore sizes.

Bokhari et al. (2007b) revealed that the way of adding inner phase into the continuous phase (syringe pump or dropping funnel) also influences the emulsion stability, the pore size distribution of the droplets, and the reproducibility. Another emulsification route; the multiple emulsion method, combines and mixes all the components from the oil and water phase together. But as the emulsion prepared using this method forms gradually, the system needs to be stirred until the HIPE forms (Richez et al., 2005).

Apart from the parameters mentioned above, it is also important to note that, every parameter that affects the energy input for the droplet breakup; such as mixing time, the batch volume of the emulsion, materials and the diameter of the emulsification container, and the magnetic stirrer/paddle size (where relevant) directly affects the mixing efficiency of the emulsion and will have an impact on the final morphology. Keeping these parameters constant between batches helps the consistency and reproducibility of the PolyHIPEs.

### HIPEs to PolyHIPEs

#### Emulsion Solidification Approaches

##### Free-radical polymerisation

PolyHIPEs based on many popular polymers, such as; acrylates, methacrylates, and styrenes are synthesised using free-radical polymerisation (FRP) (Zhang et al., 2019). The type of the initiator (section “Initiators”) used in the emulsion composition determines one of the following initiation routes of FRP; (i) thermal-initiated polymerisation, (ii) photo-initiated polymerisation, and (iii) redox-initiated polymerisation.

The earliest examples of the PolyHIPEs (during the 1980s) were based on thermal polymerisation (Donald and Zia, 1982; Jones et al., 1986). In this process, emulsion mixture that contains thermal initiator is exposed to heat in an oven or in a heat bath for 6–48 h for the polymerisation. The temperature is often kept quite high [60–70°C (Busby et al., 2002; Bokhari et al., 2005, 2007b; Christenson et al., 2007; Lumelsky et al., 2008)] to decompose the initiator into radicals thermally. Exceptionally, polypropylene fumarate dimethacrylate (PFDMA) HIPEs can be polymerised at 37°C (Moglia et al., 2011; Robinson et al., 2014). Also, by the use of a catalyst, the polymerisation temperatures can be reduced (Zhang T. et al., 2016).

In photo-initiated polymerisation, emulsions are prepared using photosensitive materials as a continuous phase and photoinitiators are exposed to light to be able to generate free-radicals and initiate the polymerisation. This method has many advantages over thermal-induced polymerisation; polymerisation takes places at room temperature (RT) just in seconds to minutes depending on the sample size. Photopolymerisation is an efficient polymerisation route, especially in relatively small samples, as ultraviolet (UV) penetration depth can be limited in a larger volume of emulsions. Photo-initiated polymerisation of the emulsions, specifically HIPEs, were described in patents in 1986 (Tanny et al., 1986) and 1999 (Thunhorst et al., 2003), respectively. A more detailed experimental procedure of photo-initiated polymerisation of HIPEs was reported by Pierre et al. (2006). However, lately, a number of studies about the development of PolyHIPEs based on photocurable materials such as thiolene (Johnson et al., 2015), meth(acrylates) of PCL (Aldemir Dikici et al., 2019a,b) or gelatin (Barbetta et al., 2006) have increased the potential applications. Redox-initiated polymerisation, which uses reducing and oxidising agents, is also another FRP route used in the polymerisation of HIPEs (Moglia et al., 2014c).

##### Step-growth (condensation) polymerisation

Integration of step-growth polymerisation into emulsion templating process has been successfully implemented in polyurethane PolyHIPEs (David and Silverstein, 2009). It is synthesised using diisocyanate and PCL triol, and the reaction of a diisocyanate with water produced urea groups and carbon dioxide (CO_2_). The bubbles of generated CO_2_ created additional porosity into PolyHIPE structure.

##### Ring-opening polymerisation

Development of PolyHIPEs by ring-opening polymerisation (ROP) of cyclic monomers is a relatively new route of polymerisation HIPEs. ε-caprolactone and L-lactide are the most widely used monomers in this approach (Pérez-García et al., 2016; Sharma et al., 2016; García-Landeros et al., 2019; Utroša et al., 2019; Yadav et al., 2019). One of the most important advantages of this technique is the elimination of diluting solvents from the emulsion composition due to the low viscosity of the cyclic monomers. Catalyst concentration is the deterministic factor of the rate and the degree of polymerisation. The polymerisation temperatures and the polymerisation durations vary between 37 and 120°C and 6 h to overnight, respectively.

##### Solvent evaporation

Although the term PolyHIPE refers to polymerised emulsions, recently polylactic acid (PLA), PCL, polylactic-co-glycolic acid (PLGA) PolyHIPEs solidified without polymerisation have been reported (Hu et al., 2014a,b, 2015, 2017; Samanta et al., 2016a; Yang et al., 2017). This process is based on dissolving the non-functional, linear, high molecular weight polymer in an appropriate diluting solvent, followed by emulsification, and finally solidification of HIPEs via solvent evaporation. As high molecular weight polymers are commercially available, this process does not require polymer synthesis or functionalisation steps. PolyHIPEs can be fabricated either with moulding or 3D printing. The main disadvantages of this technique are the long solidification process (24–48 h) (Yang et al., 2017), and the requirement of development of emulsions with high stability that would keep the shape until solidification.

##### Crosslinking

Crosslinking of PolyHIPEs as a solidification approach can be either ionic, thermal or enzymatic. Alginate PolyHIPEs has been synthesised using calcium ions (Barbetta et al., 2009; Zhou et al., 2013). This reversible gelation can be de-crosslinked using sodium citrate. Solidification of gelatin HIPEs using enzymatic crosslinking also has been reported (Barbetta et al., 2006). In another study, gelatin PolyHIPEs has been obtained by physical thermal-crosslinking at 4°C (Oh et al., 2015).

#### Fabrication Routes

##### Casting (moulding)

**FIGURE 10:**
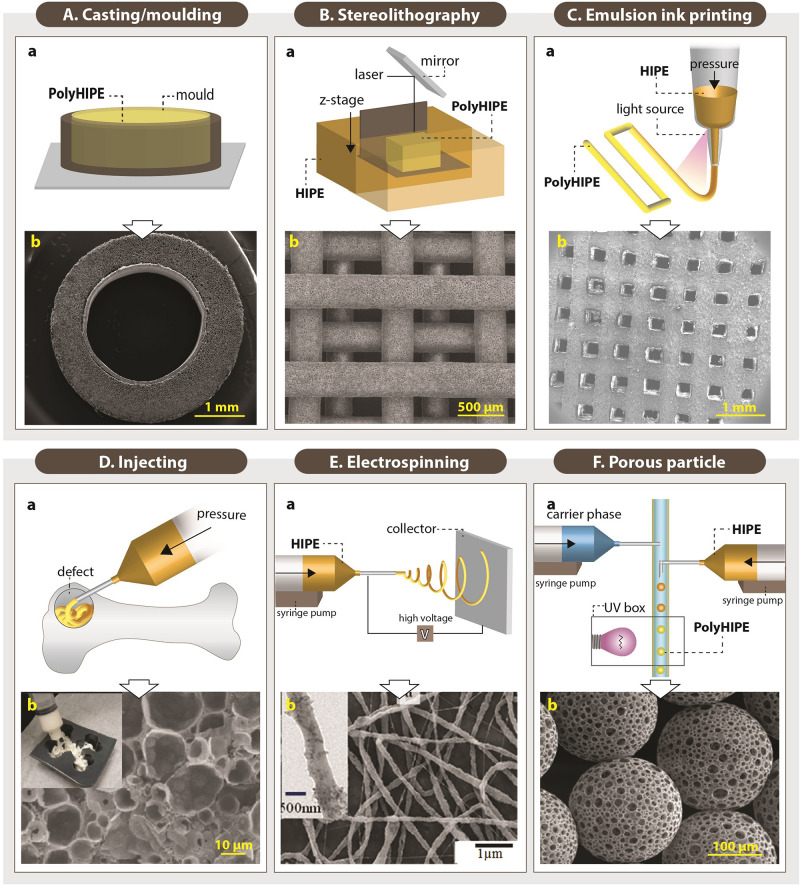
Setups of various fabrication routes of emulsion templated scaffolds **(Aa,Ba,Ca,Da,Ea,Fa)**, and scanning electron microscopy images of these scaffolds **(Ab,Bb,Cb,Db,Eb,Fb)**. (Original images were cropped, and scale bars were added to enhance the figures). Images **(Ab,Bb,Cb,Fb)** were adapted from Dikici et al. (2020a), Owen et al. (2016), Aldemir Dikici et al. (2020), and Paterson et al. (2018) respectively, under The Creative Commons License. Image **(Db)** was adapted with permission from Moglia et al. (2011), Copyright 2011 American Chemical Society. Image **(Eb)** was adapted with permission from Samanta et al. (2017a), Copyright 2017 American Chemical Society.

The earliest examples of PolyHIPE scaffolds were fabricated using the casting technique (Figure 10Aa). This technique is the easiest way of manufacturing PolyHIPE scaffolds with almost no additional technical equipment requirement. Silicone (Naranda et al., 2016; Aldemir Dikici et al., 2019a,b; Dikici et al., 2020a), polyvinyl chloride (Busby et al., 2002), polytetrafluoroethylene (Hayman et al., 2004, 2005; Lee et al., 2017), glass (Audouin et al., 2012), polypropylene (Moglia et al., 2014b), polycarbonate centrifuge tubes (Carnachan et al., 2006; Hayward et al., 2013b), and aluminium (Moglia et al., 2011) are some of the materials reported to be used to create moulds for the fabrication of PolyHIPE scaffolds. Recently, we have shown that mould material has a significant impact on the morphology of the contact surface of PolyHIPEs which have fully or partially closed cellular morphology (Aldemir Dikici et al., 2019a). Similar to our finding, the influence of the mould material on PolyHIPE morphology and HIPE stability has also been reported previously by Cameron (2005).

In order to avoid the closed-pores on the surface of the PolyHIPEs, the moulded PolyHIPE blocks are typically sectioned using various methods and benefit from the open porous cross-sectional surface. Vibratomes (Hayward et al., 2013a; Aldemir Dikici et al., 2019a) and microtomes (Bokhari et al., 2007b) allow precise micro-scale thick scaffolds to be obtained. Additionally, tabletop precision saws (Robinson et al., 2014, 2016; Whitely et al., 2017), and scalpels/razor blades (Caldwell et al., 2012; Aldemir Dikici et al., 2019a,b) have been used cut monoliths to millimetre scale sections.

Casting enables the manufacturing of scaffolds in a wide range of shapes and sizes, depending on the mould design (Paljevac et al., 2018; Diez-Ahedo et al., 2020; Dikici et al., 2020a; Owen et al., 2020). Recently, Dikici et al. (2020a) reported the fabrication of the PolyHIPEs in tubular form by designing a re-usable tubular silicone mould system that, HIPE can be injected into, polymerised, and recovered easily (Figure 10Ab). Also, sacrificial polymer beads made of PDMS (Paljevac et al., 2018) or alginate (Owen et al., 2020) have been incorporated into emulsion composition prior to polymerisation in a mould to simply introduce multiscale porosity to PolyHIPEs.

##### Stereolithography

Fabrication of TE scaffolds using AM techniques gained huge attention in the last decades due to various advantages of the AM such as enabling the manufacture of complex shapes using a broad range of materials, high reproducibility and providing control on the exterior architecture of the scaffolds. However, manufacturing of pores less than 20 μm using the current AM techniques remains a challenge (Pei et al., 2017). Alternatively, combining emulsion templating with AM techniques such as stereolithography or emulsion ink printing enables the fabrication of well-defined multiscale porous complex scaffolds.

Stereolithography is a laser-based fabrication method that selectively polymerises photo-sensitive liquid resin layer-by-layer. It provides higher accuracy compared to other AM technologies (Kawata et al., 2001; Johnson et al., 2013; Yang et al., 2020). The emulsions with low viscosity are preferable to be able to provide enough spreading on the z-stage while the building chamber is moving in the *z*-axis (Figure 10Ba).

Johnson et al. (2013) reported the 3D defined complex structures of PolyHIPEs made of EHA:IBOA using micro-stereolithography up to 30 μm accuracy. Exactly on the same date, Sušec et al. (2013) reported the development of stable photocurable thiolene HIPEs that can be used to produce PolyHIPEs using stereolithography. Fabrication of EHA:IBOA PolyHIPEs via stereolithography in wood-pile structure has been reported many times for various TE applications (Figure 10Bb; Malayeri et al., 2016; Owen et al., 2016; Wang et al., 2016). However, manufacturing of EHA:IBOA PolyHIPEs using stereolithography cause the formation of surface skin that is characterised with the closed pore at the surface. Sherborne et al. (2018) showed that the use of UV absorbers could reduce skin formation without causing any toxic effect on cells.

##### Emulsion ink printing

Emulsion ink printing is another convenient AM route to combine with emulsion templating for the fabrication of multiscale porous scaffolds. This method is based on the preparation of emulsion inks, filling the printing head reservoir with a required amount of the emulsion, and printing the emulsion in the designed 3D shape (Figure 10C). Nozzle size, pressure and printing speed are some of the parameters that have an impact on the final structures. There are two approaches used in this manufacturing technique; (i) simultaneous printing and solidification of the HIPEs and (ii) printing HIPEs and subsequent solidification. Only for the first approach, emulsions prepared from photocurable materials are needed. Otherwise, emulsion printing is not limited to use of the photocurable resins as in stereolithography.

The shear-thinning nature and the high viscosity of HIPEs make them good candidates to be used as inks for the 3D printing. It is essential to highlight the fact that emulsion viscosity is inversely proportional to the size distribution of the water droplets (Ilia Anisa and Nour, 2010). Thus, the viscosity of the emulsion should be high enough for successful printing of the emulsion inks and low enough for enabling the manufacturing of the scaffolds with a pore size range that allows cell infiltration. Recently, a few publications have reported the use of emulsion ink printing for the fabrication of bone TE scaffolds (Sears et al., 2016; Yang et al., 2017; Aldemir Dikici et al., 2020). Unlike stereolithography, emulsion ink printing enables fabrication of heterogeneous structures made of different inks by using different printing heads (Hu et al., 2019).

##### Injecting

Although AM techniques are first to come to the mind to fabricate defect matching scaffolds, their use requires highly accurate imaging techniques to be able to create 3D custom-made models (Moglia et al., 2014b). Alternatively, injectable materials that harden *in situ* can fill irregular shapes by minimal invasive delivery (Figure 10D), and they can also be used as a carrier for cells and other biological molecules (Temenoff and Mikos, 2000; Chang et al., 2017; Guyot and Lerouge, 2018).

The main prerequisites for the development of injectable emulsions are the elimination of the toxic solvents and enabling polymerisation at physiological temperatures. Thus, there is a limited number of materials that can be used to develop injectable HIPEs.

PPF HIPEs, discussed in detail in section “Monomers/Macromers,” have a suitable viscosity for injection from a syringe, and they can solidify at body temperature in 15 min. They have been shown to be stable for storage up to 6 months and to integrate into the host tissue successfully (Moglia et al., 2014c; Robinson et al., 2014). Moglia et al. also developed PCL-diisocyanate (PCL-DI) and PCL-triisocyanate (PCL-TI) based injectable PolyMIPEs without the use of any organic solvents, but curing time at 37°C has been reported as 48 h which limits their clinical applicability (Moglia et al., 2014b). Zhou et al. (2013) reported injectable alginate PolyHIPEs (not HIPEs). Following the preparation of o/w HIPEs with methacrylate functionalised alginate, they were thermally set. It was shown that PolyHIPEs could be extruded from a needle by retaining its morphology and further crosslinked using calcium ions. Similarly, Oh et al. (2015) developed injectable poly(*N*-isopropylacrylamide) (PNIPAM) grafted gelatin PolyHIPEs.

##### Electrospinning

Electrospinning is a versatile route for the fabrication of the fibres with varying diameters from nanometres to micrometres scale using a wide range of materials (Aldemir Dikici et al., 2019a; Dikici et al., 2019, 2020a,b; Mangir et al., 2019a; Figure 10E). There are many examples of electrospinning of emulsions in the literature (Mangir et al., 2016; Pal et al., 2017; Samanta et al., 2017b), but a few studies reported the electrospinning of HIPEs. Samanta et al. (2016a) reported the fabrication of the PCL electrospun fibres from HIPE. Briefly, PCL (dissolved in toluene) and polyvinyl alcohol (PVA) (dissolved in water) were used for continuous and internal phases, respectively. They showed that increasing the continuous/internal phase ratio reduces the fibre diameter. The same group also reported the electrospinning of solvent-free Pickering PCL HIPEs (Samanta et al., 2016b).

##### Porous particle manufacturing

Microporous PolyHIPE particles can be applied to the defect site by injecting and used as substrates for controlled drug delivery (Moglia et al., 2014a; Whitely et al., 2019). They can be created using multiphasic emulsion systems that are mostly water-in-oil-in-water (w/o/w) emulsions. The easiest fabrication route of porous particles is dropwise addition of w/o emulsion into the water while the system is stirred [controlled stirred-tank reactor (CSTR)]. Although CSTR is practical and does not need a complicated setup, it only enables fabrication of polydisperse particles, and it does not provide an accurate control on particle size. Recently, microfluidic systems gained attention for the fabrication of porous particles due to providing high control over particle size. The process is briefly based on injecting w/o emulsion and water phase into the tubing system using syringe pumps (Figure 10F; Gokmen and Du Prez, 2012). Bead size can be controlled by changing the nozzle size, flow rates of the water phase and emulsion phase. Paterson et al. reported that microfluidics enable the manufacturing of the beads with narrower size distribution compared to particles fabricated using CSTR (Paterson et al., 2018).

### Monomers/Macromers

#### Hydrophobic Polymers for the Fabrication of w/o PolyHIPEs

##### Non-degradable polymers

The earliest studies on the development of emulsion templated substrates used styrene as a monomer (Guenther, 1959; Barby and Haq, 1985; Lee et al., 1992a,b; Akay et al., 2000). In 1992, commercial styrene PolyHIPE microcarriers (provided by the company, Microporous Materials) were tested with suspension-growing cell lines for the production of a therapeutic protein, and they were found advantageous as being sterilisable, cheap, and suitable for surface functionalisation (Lee et al., 1992a,b). In 1993, human endothelial cells were cultured on the same microcarriers for a similar purpose, and they reported that PolyHIPEs did not support cell growth (Schrimpf and Friedl, 1993). Akay et al. (2000) have a patent on styrene/DVB PolyHIPEs as a cell growth media in 1998. Since then, styrene/DVB is one of the highest reported PolyHIPE materials in the literature.

The blend of acrylate based-monomers; EHA:IBOA is another commonly reported non-degradable material that is mostly solidified using photo-initiated polymerisation (Pierre et al., 2006; Malayeri et al., 2016; Paterson et al., 2018). Owen et al. showed that PolyHIPEs prepared by changing the ratios of EHA or IBOA yield in varying mechanical properties (up to a 60-fold change) (Owen et al., 2015, 2016).

##### Degradable polymers

###### PCL

**FIGURE 11:**
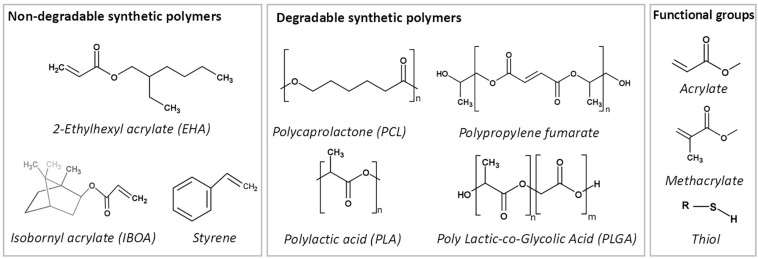
Commonly used synthetic polymers to prepare emulsion templated matrices.

As TE scaffolds are desired to be made of biodegradable materials, the development of biodegradable PolyHIPEs is important to satisfy the need of implantable TE constructs (Figure 11). PCL is the earliest biodegradable polymer that has been included in PolyHIPE composition. However, the development of HIPEs made of PCL has been problematic over the years due to the high viscosity of the polymer, which limits the mixing of two phases during emulsification (Busby et al., 2001; Lumelsky et al., 2008, 2009; Lumelsky and Silverstein, 2009).

The earliest reported PolyHIPE made from PCL was created by copolymerisation of PCL diacrylate with non-degradable monomers (Busby et al., 2001). Various diluting solvents were included in HIPE composition to reduce the viscosity of PCL (Busby et al., 2001; David and Silverstein, 2009; Changotade et al., 2015). Johnson et al. reported the incorporation of 76% PCL triacrylate into HIPE composition when DCE used as a porogenic solvent (Johnson et al., 2015). Recently, we have reported the manufacturing route of PolyHIPEs fully made of photocurable PCL tetra-methacrylate diluted by a solvent blend of chloroform and toluene (Aldemir Dikici et al., 2019b). Also, recently, the development of high molecular weight non-reactive PCL PolyHIPEs has been reported by solvent evaporation (Hu et al., 2015, 2017; Samanta et al., 2016a; Yang et al., 2017).

###### PLA and PLGA

PLA and its copolymer PLGA are widely used biomaterials for fabrication of TE scaffolds. Busby et al. (2002) reported the development of thermal polymerised PLA diacrylate PolyHIPEs (PLA content up to 40%) by diluting the oil phase with methyl methacrylate (MMA). Also, synthesis of PolyHIPEs based on PCL and PLA blends, without the use of any diluents, via ring opening polymerisation also has been reported (Pérez-García et al., 2016). Recently, Hu et al. reported the development of composite HA/PLA (Hu et al., 2014b) and HA/PLGA (Hu et al., 2014a) scaffolds by Pickering emulsion templating and solvent evaporation.

###### PPF

PPF, an unsaturated linear polyester, can be easily cured through double-bound on the backbone of the fumarate using various crosslinking agents (He et al., 2001). It is commonly suggested to be cured by *in situ* crosslinking in the defect site (Suggs et al., 1999). Its degradation products are nontoxic monomers (He et al., 2001; Lee et al., 2006). Due to its aforementioned advantages, it has been used in various biomedical applications, including TE scaffolds and orthopaedic implants (Kasper et al., 2009). Fumarate based PolyHIPEs [PPF (Christenson et al., 2007), PFDA (Christenson et al., 2007) and PFDMA (Moglia et al., 2011)], are groups of the well-established biodegradable PolyHIPE compositions.

Christenson et al. (2007) developed PPF PolyHIPEs that can be cured at 60°C in 48 h in the presence of PFDA as a crosslinker. They showed the tunability of the material by changing PPF, PFDA and toluene concentrations. Later on, Moglia et al. (2011) reported the development of injectable solvent-free PFDMA PolyHIPE that can be cured at 37°C. However, the structures obtained exhibited closed cellular morphology. Robinson et al. (2014) hypothesised that including an oil-soluble initiator into PFDMA PolyHIPE might induce organic phase initiation and this results in open porous monoliths. Indeed, they have used both an oil-soluble initiator; BPO and a water-soluble initiator, APS, and PolyHIPEs with BPO showed open-porosity. However, the curing time of the HIPEs was still long (overnight) for the ultimate aim of *in situ* crosslinking (Robinson et al., 2014). Moglia et al. used the redox initiated polymerisation rather than thermal initiation in order to reduce the curing time. They created two PolyHIPE compositions; one with BPO as an initiator and other with trimethylaniline (TMA) as reducing agent and used a syringe with the double barrel for the injection of the emulsion and enabled polymerisation of HIPEs just in 15 min (Moglia et al., 2014c).

###### Thiol (ene/yne)

Thiol(ene/yne) chemistry [also classified as click chemistry (Hoyle and Bowman, 2010)] is the reaction between a thiol and an alk(ene/yne) to thioether. This high yield reaction has recently gained attraction in various applications, including the development of thiol(ene/yne) PolyHIPEs (Lovelady et al., 2011; Caldwell et al., 2012; Johnson et al., 2015).

Lovelady et al. (2011) reported the development of thiol(ene/yne) PolyHIPEs. In the follow-up study, Caldwell et al. developed TMPTA and dipentaerythritol penta/hexa-acrylate (DPEHA)-based thiolene PolyHIPEs and showed the dependency of mechanical properties to monomer selection (Caldwell et al., 2012). Johnson et al. (2015) reported the development of photocurable PCL triacrylate thiolene PolyHIPEs with up to 95% interconnected porosity. The degradation products have been shown to be non-toxic on fibroblasts up to a concentration of 0.1 mg/mL. Whitely et al. also developed thiolene PolyHIPEs made of tetra-functional thiol, pentaerythritol tetrakis-3-mercaptoproprionate, and PFDMA (Whitely et al., 2017), and they showed the hydrolytic and accelerated degradation profiles of these scaffolds.

#### Hydrophilic Polymers for the Fabrication of o/w PolyHIPEs

In 1997, Naotaka Kitagawa described the development of hydrophilic PolyHIPEs (Kitagawa, 2001). Since then, a number of naturally derived polymers have been used to fabricate PolyHIPEs from o/w emulsions. These matrices have the advantages of being hydrophilic, biocompatible and biodegradable, often similar to extracellular matrix (ECM) components to be used as materials for the fabrication of TE scaffolds. However, they have the disadvantages of having a high degree of batch-to-batch variability and comparably lower mechanical strength than synthetic counterparts (Cheung et al., 2007; Shoichet, 2010).

Gelatin is one of the most commonly used natural biopolymers for the fabrication of TE scaffolds. As it is derived from collagen of skin, bone or tendon of animals, it is highly abundant and cost-effective (Hoque et al., 2015). In 2005, Barbetta et al. successfully developed gelatin-methacrylate PolyHIPEs with up to 95% internal phase using FRP (Barbetta et al., 2005b). Following this, they also reported the development polysaccharides; dextran and pullulan methacrylate PolyHIPEs (Barbetta et al., 2005a). They also developed gelatin PolyHIPEs that are solidified via enzymatic crosslinking (Barbetta et al., 2006). Although PolyHIPEs obtained via FRP of gelatin exhibited better-defined morphology, enzymatically crosslinked PolyHIPEs were found less toxic on hepatocytes and showed an improved expression of adhesion proteins (Barbetta et al., 2006). Oh et al. (2015) developed gelatin PolyHIPEs by grafting gelatin with poly(*N*-isopropylacrylamide) (PNIPAM). Due to the amphiphilic nature of gelatin-graft-PNIPAM as a continuous phase, they managed to incorporate an internal phase of up to 90% without the use of any surfactants. Recently Yuan et al. (2019) reported the fabrication of gelatin PolyHIPEs with 92% porosity by two-step crosslinking and freeze-drying. Alginate, a polysaccharide derived from seaweed, is another biomaterial that can be used to fabricate PolyHIPE scaffolds (Barbetta et al., 2009; Zhou et al., 2013). Krajnc et al. (2005) also reported the development of o/w HIPEs from a synthetic hydrophilic monomer, acrylic acid.

### Post-processes

#### Improving the Biomimetic Behaviour of the PolyHIPE Scaffolds

The suitability of the morphology of PolyHIPE matrices to be used as TE scaffolds has been well-accepted. However, as PolyHIPEs are most commonly created using w/o emulsions, they are highly hydrophobic, and lack of functionality which limits their interaction with biological tissues (Richardson et al., 2019). Thus, starting from the early 2000s, researchers started to explore the ways of enhancing the biological activities of the PolyHIPE scaffolds using various methods such as chemical functionalisation (Aldemir Dikici et al., 2019a), incorporation of the hydrophilic particles such as HA (Akay et al., 2004; Wang et al., 2016), incorporation of a single biologically active agent (Ratcliffe et al., 2019; Richardson et al., 2019), or decoration PolyHIPEs with cell-derived *in vitro* generated ECM (Aldemir Dikici et al., 2020; Table 2).

**TABLE 2:** Various functionalisation strategies for PolyHIPE scaffolds from the literature.

**Approach**	**Monomer/macromer**	**Improvement**	**Results**	**References**
Chemical functionalisation/Incorporation of functional monomers	Styrene, divinylbenzene, and 2-ethylhexyl acrylate	Incorporation of the monomer acrylic acid into the water phase of w/o emulsion	7.5% carboxylic acid functionality, Increased wettability, No adverse effect on cell attachment	Hayward et al., 2013b

Chemical functionalisation/Post-functionalisation	Poly(styrene/ethylene glycol dimethacrylate)	Air plasma treatment	Increased wettability, Enhanced cell attachment	Pakeyangkoon et al., 2012
	2-Ethylhexyl acrylate and isobornyl acrylate	Air plasma or acrylic acid plasma treatment	Enhanced cell attachment and cellular metabolic activity	Owen et al., 2016
	Polycaprolactone tetramethacrylate	Air plasma treatment	Increased wettability, Enhanced cell infiltration	Aldemir Dikici et al., 2019a

Incorporation of ceramic particles	Styrene	Hydroxyapatite/internal phase	Higher cell viability, penetration and osteoblast differentiation	Akay et al., 2004; Bokhari et al., 2005
	2-Ethylhexyl acrylate and isobornyl acrylate	Hydroxyapatite/internal phase	Improved tensile modulus	Wang et al., 2016
	Thiol-acrylate	Hydroxyapatite and strontium-modified hydroxyapatite/continuous phase	Improved cell adhesion and proliferation	Lee et al., 2017
	Poly fumarate dimethacrylate	Calcium phosphate, hydroxyapatite/demineralised bone matrix	Improved cell attachment andproliferation, Enhanced osteogenicand angiogenic activity	Robinson et al., 2016

Incorporation of biomolecules	Styrene	Peptide coating (Physical)	Improved osteoblast penetration depth and the alkaline phosphatase activity	Bokhari et al., 2005
	Styrene	Poly-D-lysine & laminin coating (Physical)	Poly-D-lysine & laminin coating was found advantageous over only Poly-D-lysine	Hayman et al., 2005
	Thiolene	Fibronectin coating (Physical)	Improved cell attachment, proliferation and infiltration	Eissa et al., 2018
	Thiol-acrylate	Maleimide-derivatised RGD peptide attachment	Improved cell attachment and proliferation	Richardson et al., 2019
	Polycaprolactone tetramethacrylate	*In vitro* cell-derived extracellular matrix deposition	Improved cell attachment and proliferation, Enhanced osteogenic and angiogenic activity	Aldemir Dikici et al., 2020

Chemical functionalisation/Incorporation of functional monomers + Incorporation of biomolecules	Thiol-acrylate	Functionalisation with sulfo-SANPAH + Covalent fibronectin attachment	Improved cell attachment and infiltration	Richardson et al., 2019
	Styrene	Incorporation of pentafluorophenyl acrylate into the oil phase of the HIPE + Galactose attachment	Higher albumin synthesis by hepatocytes	Hayward et al., 2013a

##### Chemical functionalisation

The surface of the scaffolds can be modified to create functional groups that act as hooks for biomolecules and cells. Amines, hydroxyl, carbonyl, carboxyl, epoxy groups, and thiols are the functional groups generally used to improve cell-material interaction or enabling the incorporation of other biomacromolecules into the scaffolds (Richbourg et al., 2019). A wide range of techniques can be used for chemical functionalisation of PolyHIPEs (Kircher et al., 2013a). There are two main approaches for chemical functionalisation of PolyHIPEs; (i) incorporating co-monomers with desired functionality into HIPE composition and (ii) post-functionalisation of PolyHIPEs. Although the first approach seems convenient as the functionality can be improved using a one-step route, incorporating hydrophilic monomers may cause destabilisation of the emulsion, results in bigger pores, and less well-defined morphology (Kircher et al., 2013b). The second approach enables the introduction of functional groups without changing the morphology of the PolyHIPEs.

Hayward et al. incorporated acrylic acid into the water phase of the styrene/DVB/EHA PolyHIPE, and they verified the success of the carboxylic acid functionalisation by X-ray photoelectron spectroscopy, wettability analysis, and toluidin blue staining (Hayward et al., 2013b).

Post-polymerisation of thiol-acrylate PolyHIPEs has also been reported. During the polymerisation of thiol-acrylate, there are two competing addition reactions that occur; the first one is between thiols and acrylates, and the second one is between acrylates and acrylates. With a stoichiometric thiol to acrylate ratio, the occurrence of the second reaction will result in the presence of unreacted residual thiols. These can be used for further functionalisation using various reactions, such as thiol-ene click chemistry (Langford et al., 2014) or Michael addition reaction (Ratcliffe et al., 2019).

Plasma treatment is one of the most common and effective ways of post-functionalisation to promote hydrophilicity of the polymer surfaces by adding polar groups to the surface of the material without altering the bulk properties (Jokinen et al., 2012; Abedalwafa et al., 2013; Valence et al., 2013; Shafei et al., 2017; Ivanova et al., 2018). Owen et al. showed that both air and acrylic acid plasma treatment improved the attachment and proliferation of mesenchymal progenitors on acrylate-based PolyHIPEs, whereas untreated scaffolds did not support cell attachment (Owen et al., 2015, 2016). Pakeyangkoon et al. reported that water contact angle on poly(styrene/ethylene glycol dimethacrylate) PolyHIPE dramatically dropped and attachment of fibroblast-like cells on PolyHIPEs improved after air plasma treatment of the scaffolds (Pakeyangkoon et al., 2012). Recently, we reported that air plasma treatment improved the wettability of highly hydrophobic polymer, PCL PolyHIPEs, and it enhanced infiltration of bone cells through PolyHIPE scaffolds (Aldemir Dikici et al., 2019a).

##### Incorporation of ceramic particles

Incorporating HA, a bioceramic that is present in native bone with a percentage of 70%, within a scaffold is a common approach to improve the biocompatibility, osteoconductivity, and osteoinductivity of polymer-based bone TE scaffolds. Although emulsions are metastable systems that are readily destabilised by incorporation of additional particles, many researchers have managed to incorporate nano/micro HA particles into the various PolyHIPE compositions to improve the biological or mechanical properties of PolyHIPEs rather than using HA as Pickering particle.

HA is commonly included in HIPE composition before emulsification. It can be added either into the oil phase or into the water phase. Akay and Bokhari et al. incorporated commercially available HA into the water phase of the w/o PolyHIPEs to be able to locate the HA particles only on the surface of the pores. They showed that 0.5% HA (of the aqueous phase) incorporated DVB/styrene PolyHIPE increased the viability of cells, cell penetration into the scaffolds, and osteoblast differentiation *in vitro* (Akay et al., 2004; Bokhari et al., 2005). Wang et al. (2016) incorporated 4–32% HA that was synthesised *in house* into the water phase of the EHA:IBOA PolyHIPE. No pore size difference was observed between groups except that 32% HA incorporated HIPEs showed reduced stability and increased pore size. However, the tensile modulus of this group was increased more than twofold in comparison to the control group, probably due to having bigger pore sizes. Lee et al. (2017) incorporated (5–10%) HA and strontium-modified HA into a PolyHIPE composition by adding it into the oil phase of the emulsion. HA incorporation increased the pore size distribution with increasing concentration and significantly increased the compressive strength. Although it was incorporated into the oil phase, SEM/Energy Dispersive X-Ray Analysis (EDX) images showed the presence of HA particles on the surface of the scaffolds. Incorporation of HA, particularly strontium-modified HA, increased cell adhesion and proliferation when compared to unmodified PolyHIPE. Similarly, Robinson et al. incorporated 2 wt% calcium phosphate nanoparticles, 5 wt% HA nanoparticles, and 15 wt% demineralised bone matrix (DBM) particles into injectable PFDMA HIPEs (Robinson et al., 2016). Particles affected neither the emulsion stability nor the pore size of the PolyHIPEs. Similar to the results of Lee et al., although the particles were added into the oil phase of the emulsion, transmission electron microscopy (TEM) images showed the localisation of the particles on the surface unless they aggregated.

##### Incorporation of biomolecules

Incorporation of the biomolecules into the composition of PolyHIPE scaffolds requires moderate operation conditions in terms of temperature and solvents. Biomolecules such as peptides and ECM proteins can be either covalently attached to the surfaces or physically absorbed/coated to the PolyHIPEs.

Robinson et al. applied biologically inspired self-assembling peptide hydrogel into HA-doped styrene PolyHIPEs via cell seeding suspension. Osteoblast penetration depth and the alkaline phosphatase (ALP) activity have been shown to be increased in comparison with the control (Bokhari et al., 2005). Hayman et al. used Poly-D-lysine and laminin coating on DVB/styrene PolyHIPEs and showed the advantages of Poly-D-lysine and laminin coating over only Poly-D-lysine coating in terms of increasing the mean neurite length (Hayman et al., 2004, 2005). Eissa et al. (2018) reported that fibronectin coating of thiolene PolyHIPEs significantly increased the attachment, proliferation and infiltration of primary human endometrial epithelial and stromal cells when compared to the uncoated PolyHIPEs.

Richardson et al. functionalised thiol-acrylate PolyHIPE scaffolds with covalent attachment of fibronectin using a two-step procedure. First, they functionalised PolyHIPEs with *N*-sulfosuccinimidyl-6-(4′-azido-2′-nitrophenylamino hexanoate (sulfo-SANPAH)), which is a photo-linker compound that enables conjugation of biomolecules to the surfaces, and then they further functionalised the surface by covalent attachment of fibronectin to sulfo-SANPAH molecule. An improved cell attachment and infiltration of human endometrial stromal cells have been found when compared to unmodified, just sulfo-SANPAH functionalised, and fibronectin-absorbed (physically) PolyHIPEs (Richardson et al., 2019).

Ratcliffe et al. functionalised thiol-acrylate PolyHIPE using maleimide-derivatised cyclo-arginine-glycine-aspartate (RGD) peptide by benefiting from the reaction between the unreacted thiols in PolyHIPE and the maleimide. While non-functionalised scaffolds did not support attachment and proliferation of human embryonic stem cells, PolyHIPEs functionalised with RGD showed significantly higher proliferation and infiltration rate (Ratcliffe et al., 2019).

Hayward et al. incorporated pentafluorophenyl acrylate (PFPA) into the oil phase of the styrene HIPE to be able to conduct a coupling reaction between ester groups of PFPA and galactose afterward, as hepatocytes are known to have specific receptors that bind to galactose. Hepatocytes have been shown to proliferate on the functionalised scaffold, and they showed significantly higher activity on galactose functionalised PolyHIPEs in terms of albumin synthesis compared to cells cultured on unmodified PolyHIPEs (Hayward et al., 2013a).

In our recent study, we decorated 3D printed PCL PolyHIPE scaffolds with *in vitro* cell generated bone ECM rather than a single biologically active agent (Aldemir Dikici et al., 2020). This collagen and mineral-rich ECM coating was shown to improve attachment and proliferation of human mesenchymal progenitor cells (hES-MPs). Both angiogenic and osteogenic activities of biohybrid scaffolds were found to be significantly higher than the activities of the non-coated PolyHIPEs (Aldemir Dikici et al., 2020).

#### Washing

Following the fabrication of the emulsion templated scaffolds, typically a series of washing steps need to be applied to remove uncured material and residual surfactant. Insufficient washing of scaffolds may cause a toxic effect on cells. Also, they may give false colour changes on colourimetric cell viability assays such as MTT (3-[4, 5-dimethylthiazol-2-yl]-2, 5 diphenyl tetrazolium bromide) and resazurin reduction. The washing process can be conducted by either series of manual soakings in selected solvents or using Soxhlet extractor.

The solubilities of the materials that need to be removed should be considered for the selection of the washing solvent. Acetone is one of the commonly used solvents for washing of PolyHIPEs due to the high solubility of a wide range of polymers in acetone (Caldwell et al., 2012; Hayward et al., 2013a; Richardson et al., 2019). In our recent studies, we have used methanol instead of acetone due to it being less toxic and less destructive to crosslinked monoliths (Aldemir Dikici et al., 2019a,b). There are also studies reported using different solvents such as isopropanol (Bokhari et al., 2005) or combinations of multiple solvents (Krajnc et al., 2005; Zhou et al., 2013).

A limited number of studies have reported the effect of the washing method, duration and the choice of solvent on the features of PolyHIPE. Pakeyangkoon et al. showed that duration of solvent extraction has an impact on the surface area and mechanical properties of the PolyHIPEs (Pakeyangkoon et al., 2008). While an extraction time of between 6 h and 12 h improves the surface area and mechanical properties compared to non-extracted samples, mechanical properties become poorer than control when the extraction time is longer than 12 h.

#### Sterilisation

TE scaffolds should be free of contamination by living organisms such as bacteria and viruses for *in vitro* and *in vivo* tests and also for implantation to the human body. There are various methods used for this purpose, such as treatments with heat (Fleith et al., 2005; Yoganarasimha et al., 2014), gamma irradiation (Fleith et al., 2005), UV (Andrews et al., 2007), plasma (Poncin-Epaillard and Legeay, 2003; Griffin et al., 2018), ethylene oxide (Andrews et al., 2007; Yoganarasimha et al., 2014), ethanol (Griffin et al., 2018), and peracetic acid (Yoganarasimha et al., 2014). As the efficiency of the methods in terms of the degree of removal/inactivation of microorganism varies, it might be appropriate to clarify the difference between the terms of disinfection and sterilisation. While disinfection reduces the number of organisms present, this method cannot provide removal of all microorganisms, including spores. However, sterilisation indicates the removal of all kind of microorganisms including spores (Lerouge and Simmons, 2012). Most common sterilisation techniques in the clinics are ethylene oxide, gamma irradiation, and heat treatment. However, some of these methods have been found to cause compositional changes in the biomaterials (Fleith et al., 2005; Rogers, 2012; Dai et al., 2016).

Ethanol and UV treatment are commonly used for inactivation of the microorganisms on biomaterials for *in vitro* applications. However, ethanol treatment cannot inactivate bacteria spores, non-enveloped viruses, and prions. UV treatment works by damaging the DNA of microorganisms, and the major drawback of this technique is the limited penetration depth of UV. In addition, it was also found to be insufficient for inactivation of mycobacteria, bacteria spores, non-enveloped viruses, and prions. Thus, ethanol and UV treatments are categorised as medium level inactivation methods (Dai et al., 2016).

The sterilisation/disinfection method of the scaffolds should be selected by considering the material properties, application type, and experiment duration. Inactivation of microorganism on PolyHIPE scaffolds was commonly reported by using ethanol (Hayman et al., 2005; Caldwell et al., 2012; Moglia et al., 2014c; Eissa et al., 2018). There are also several studies that reported the use of UV irradiation (Moglia et al., 2011; Oh et al., 2015), gamma-irradiation (Yang et al., 2017), electron-beam irradiation (Hu et al., 2019), and autoclave (Naranda et al., 2016; Paljevac et al., 2018). Future studies investigating the effect of sterilisation methods on physical, chemical, and mechanical properties of emulsion templated scaffolds are needed to establish a greater degree of understanding of this matter.

## PolyHIPEs in TE Applications

### *In vitro* Models

In 2018, 3.53 million procedures involving living animals were conducted in the United Kingdom, and 56% of these procedures were for basic research purposes (Home Office, 2018). Although animal models are the gold standard due to their better ability to mimic complex human physiology, the 3R approach; replacing, reducing and refining of animal-based tests, should also be considered where possible (Peric et al., 2015). As an alternative to these *in vivo* platforms, the use of *in vitro* models has gained attention in various research areas such as; testing new drugs, studying diseases and monitoring of the natural behaviour of the cells at different scales (Owen and Reilly, 2018). *In vitro* models aim to mimic the natural environment of the cells isolated from the body in architectural, mechanical and biological aspects to be able to encourage cells to behave the similar way as they would behave in their own niche *in vivo*.

Cells populated in 2D tissue culture plates (TCPs) are known not to be a good representative of the *in vivo* environment of the cells. Cells grown in 2D have shown to have flattened morphology opposed to their stretched 3D morphology *in vivo*, and they have been reported to have less similar gene expression profiles to that observed *in vivo* (Abbott, 2003).

Non-degradable or slow-degrading 3D emulsion templated substrates are attractive *in vitro* test platforms due to their tunability in the physical and mechanical properties for different applications, ease of fabrication, reproducibility, and stability for long term experiments (Eissa et al., 2018).

Styrene PolyHIPEs have been used as an *in vitro* platform by many researchers. Hayman et al. proposed the differentiation behaviour of human pluripotent stem cell-derived neurons to be studied *in vitro* on styrene PolyHIPEs (Hayman et al., 2004, 2005). Bokhari et al. showed that styrene PolyHIPEs showed better results over TCP in terms of cell viability, ALP activity, and osteocalcin secretion of MG63 osteoblast-like cells, and better represents an *in vivo* environment (Bokhari et al., 2007b). The same group also cultured hepatic carcinoma cells on polystyrene PolyHIPE and proposed this system to be used as *in vitro* platform to study toxicity and screening of drugs (Bokhari et al., 2007a). Similarly, Sun et al. (2019) used styrene PolyHIPEs as a 3D tissue model to study the cytotoxicity of the cigarette smoke. Polystyrene PolyHIPE is also commercially available (Alvetex^®^). Costello et al. developed a multi-layered skin equivalent on these scaffolds and suggested its use for disease modelling and testing of cosmetics’ active compounds (Costello et al., 1993).

Non-degradable EHA and IBOA PolyHIPEs were also shown to support bone cell proliferation (Malayeri et al., 2016; Sherborne et al., 2018) and osteosarcoma growth (Malayeri et al., 2016), and they were suggested as an *in vitro* platform to study tumour tissue (Malayeri et al., 2016). Eissa et al. proposed DPEHA and trimethylolpropane tris(3-mercaptopropionate) (trithiol) PolyHIPEs as an *in vitro* model that could mimic native human endometrial architecture and function (Eissa et al., 2018).

**FIGURE 12:**
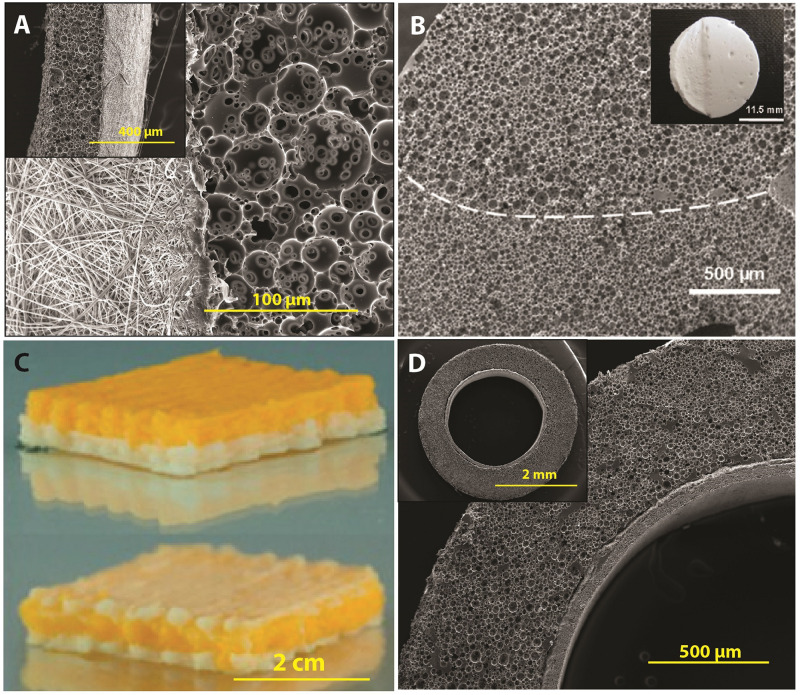
Hybrid PolyHIPE scaffolds with multiple layers: **(A)** emulsion templating combined with electrospinning for development of a membrane for guided bone regeneration, **(B)** PolyHIPEs with two different morphologies, **(C)** 3D printing of drug-loaded and drug-free HIPEs selectively, and **(D)** emulsion templating combined with electrospinning in a tubular form for the development of *in vitro* angiogenesis model. Images **(A,D)** were reproduced with permission from Ref. Aldemir Dikici et al. (2019a) and Dikici et al. (2020a), respectively, under The Creative Commons License. Image **(B)** was adapted with permission from Langford et al. (2015), Copyright 2015 John Wiley and Sons. Image **(C)** was adapted with permission from Yang et al. (2017), Copyright 2017 American Chemical Society.

Severn et al. (2019) revealed that functionalised thiolene PolyHIPEs are promising platforms to mimic the bone marrow niche. Recently, Dikici et al. developed a 3D dynamic *in vitro* model using tubular PCL PolyHIPEs combined with electrospun PCL tubes that can be used for the testing of angiogenic agents (Figure 12D; Dikici et al., 2020a). In the developed dynamic system, PCL PolyHIPE was found to be a suitable environment for comparison of the infiltration capacity of endothelial cells in response to different pro-angiogenic factors.

### PolyHIPEs as TE Scaffolds

Tissues in the body are subjected to various mechanical forces including compression, tension, torsion, and bending, and have some mechanical features such as; Young’s modulus, toughness, elasticity, tensile, and compressive strength. These mechanical features vary depending on tissue type and function. Mechanical properties of the scaffolds are required to match with the mechanical properties of the host tissue to avoid over/under mechanical loading and undesirable, heterogeneous stress distribution. The required Young’s modulus of scaffolds are reported to be in the range of 10–1,500 MPa and 0.4–350 MPa for hard and soft tissues, respectively (Hollister, 2005). Also, cells can sense and respond to the mechanical forces in their microenvironments (mechanosensitivity). Thus, the elasticity of the surface that cells are attached to is also known to affect cell behaviour, such as differentiation to specific phenotypes (Bershadsky et al., 2003; Engler et al., 2006).

#### PolyHIPEs for Hard TE

In hard TE, it is highly desirable to fabricate porous scaffolds with adequate strength and Young’s Modulus. Thus, PolyHIPE scaffolds made from synthetic polymers are preferable over naturally sourced polymers for hard TE applications due to their comparatively higher mechanical strength.

Akay et al. (2004) showed the biocompatibility of HA incorporated DVB-styrene scaffolds up to 35 days using primary rat osteoblasts. They penetrated up to 1.4 mm, differentiated and formed mineralised matrix (Akay et al., 2004).

**FIGURE 13:**
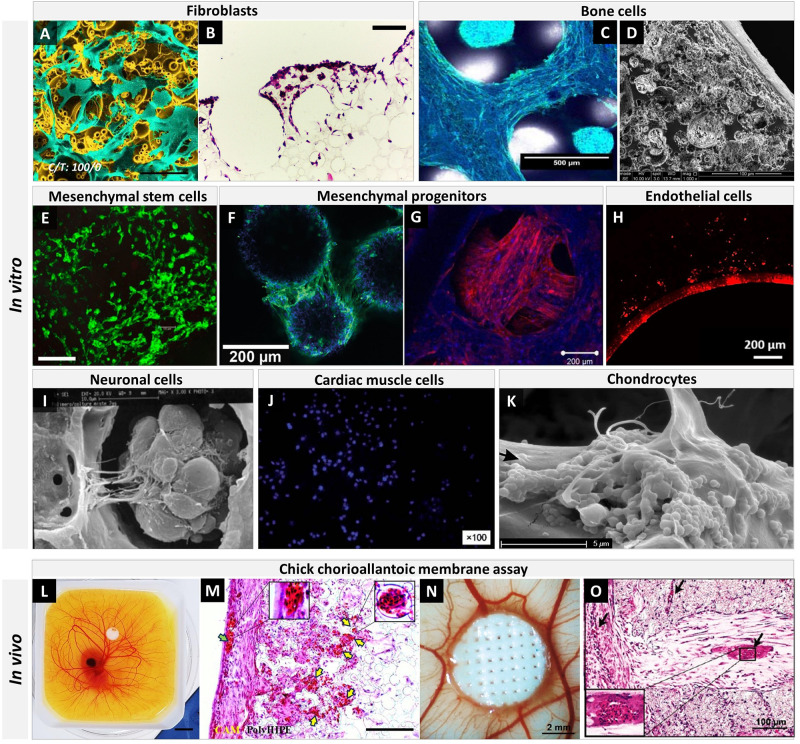
Tissue engineering applications of emulsion templated scaffolds. **(A)** False coloured scanning electron microscopy (SEM) image of human dermal fibroblasts on PCL PolyHIPE (Aldemir Dikici et al., 2019b) (scale bar: 250 μm), **(B)** H&E image of L929 fibroblasts on thiolene PolyHIPE (Johnson et al., 2015) (scale bar: 200 μm), **(C)** confocal microscopy image of MG63 bone cells on EHA:IBOA PolyHIPE (DAPI and Phalloidin-FITC) (Malayeri et al., 2016) (scale bar: 500 μm), **(D)** SEM image of murine long-bone osteocytes (MLO-A5s) on PCL PolyHIPE (Aldemir Dikici et al., 2019a) (scale bar: 100 μm), **(E)** confocal microscopy image of mouse bone mesenchymal stem cells (mBMSCs) on PCL PolyHIPE (Calcein-AM staned) (Yang et al., 2017) (scale bar: 200 μm), **(F)** confocal microscopy image of human embryonic stem cell-derived mesenchymal progenitor cells (hES-MPs) on EHA:IBOA PolyHIPE (DAPI and Phalloidin-FITC) (Paterson et al., 2018) (scale bar: 200 μm), **(G)** confocal microscopy image of hES-MPs on EHA PolyHIPE (DAPI and Phalloidin-TRITC) (Owen et al., 2016) (scale bar: 200 μm), **(H)** fluorescent microscopy image of human aortic endothelial cells (HAECs) on PCL PolyHIPE (Phalloidin-TRITC) (Dikici et al., 2020a) (scale bar: 200 μm), **(I)** SEM image of mix nerve cells (extracted from mice retina) on dextran PolyHIPE (Barbetta et al., 2005a) (scale bar: 10 μm), **(J)** fluorescent microscopy image of cardiac muscle cells (H9c2s) on polyacrylamide PolyHIPE (DAPI) (Luo et al., 2015a), **(K)** SEM image of human articular chondrocytes on polyester PolyHIPE (Naranda et al., 2016) (scale bar: 5 μm), **(L)** PCL PolyHIPE on chick chorioallantoic membrane (CAM) (Aldemir Dikici et al., 2019a) (scale bar: 10 mm), **(M)** H&E image of PCL PolyHIPE on CAM at day 14 (green arrow indicates the blood vessel on the CAM itself; yellow arrows indicate the blood vessels in PCL PolyHIPE (Aldemir Dikici et al., 2019a) (scale bar: 100 μm), **(N)** *in vitro* bone ECM decorated 3D printed PCL PolyHIPE on CAM (Aldemir Dikici et al., 2020) (scale bar: 2 mm), **(O)** H&E image of *in vitro* bone ECM decorated 3D printed PCL PolyHIPE on CAM at day 14 (black arrows indicate the blood vessels) (Aldemir Dikici et al., 2020) (scale bar: 100 μm). Images were reproduced with permission from the indicated references. Images **(A,C,D,F,G,H,K)** were adapted from Aldemir Dikici et al. (2019b), Malayeri et al. (2016), Aldemir Dikici et al. (2019a), Paterson et al. (2018), Owen et al. (2016), Dikici et al. (2020a), and Naranda et al. (2016), respectively, Images **(L,M)** were adapted from Aldemir Dikici et al. (2019a), images **(N,O)** were adapted from Aldemir Dikici et al. (2020), under The Creative Commons License. The image **(B)** was adapted with permission from Johnson et al. (2015), Copyright 2015 Royal Society of Chemistry. Image **(E)** was adapted with permission from Yang et al. (2017), Copyright 2017 American Chemical Society. The image **(I)** was adapted with permission from Barbetta et al. (2005a), Copyright 2005 John Wiley and Sons. Image **(J)** was adapted with permission from Luo et al. (2015a), Copyright 2015 Royal Society of Chemistry.

We have recently investigated the potential use of PCL PolyHIPE scaffolds for guided bone regeneration (Figure 12A; Aldemir Dikici et al., 2019a). We showed that murine long-bone osteocytes (MLO-A5s) attached, proliferated and infiltrated throughout the interconnects of the PCL PolyHIPE scaffolds. Suitability of the pores for blood vessel ingrowth was also shown using chick chorioallantoic membrane (CAM) assay (Figures 13L–O; Aldemir Dikici et al., 2019a, 2020).

Moglia et al. developed injectable PFDMA PolyHIPEs with an average compressive modulus and strength of 33 and 5 MPa and showed up to 95% initial cytocompatibility of fibroblasts after 24 h (Moglia et al., 2011). Whitely et al. (2018) have developed an *in situ* cell seeding approach for 3D printed PFDMA HIPEs to be used as bone regeneration strategy. They successfully showed the homogeneous seeding of human mesenchymal stem cells (hMSCs) all over the scaffold. HMSCs on scaffolds were mineralised and showed higher ALP activity compared to hMSCs on TCP.

Langford et al. (2015) reported the fabrication of bilayer thiol−acrylate PolyHIPEs made of two different HIPE compositions. They obtained PolyHIPE structures with different morphologies and suggested their use as scaffolds for the tissues that require layered designs such as ligaments, tendons, and bone attachments (Figure 12B).

Naranda et al. (2016) developed thiolene PolyHIPEs for cartilage regeneration and showed that PolyHIPE scaffolds fully degraded via accelerated degradation and lost 55% of their weight in PBS in 4 weeks. The Young’s modulus of the scaffolds was measured as 0.15 MPa as prepared and 0.18 MPa after 20-day culture of primary human chondrocytes on the scaffolds. Collagen type-II deposition and gene upregulation were shown using immunostaining and PCR, respectively.

#### PolyHIPEs for Soft TE

The two main components of soft tissues, such as skin, nerve, fascia, and blood vessels, are elastin and collagen, which both of them have very high water content (De Santis et al., 2004). Thus, hydrogels are preferable candidates to be used as scaffold materials for soft TE (Landers et al., 2002).

Barbetta et al. reported that dextran PolyHIPEs supports penetration and colonisation of the neurons into the inner cavities of the scaffold (Barbetta et al., 2005a). Murphy et al. showed that TMPTA, 1,6-hexanediol diacrylate (HDDA) and PEGDA thiolene PolyHIPEs support proliferation, differentiation and infiltration of induced pluripotent stem cell (iPSC)-derived human neural progenitor cells (hNPCs) (Murphy et al., 2017). Especially the PEGDA PolyHIPE was found to be a favourable substrate for hNPCs culture due to the similarity of its mechanical properties to the native human brain. Recently, the same group further explored the ability of laminin-coated PEGDA PolyHIPE for the culture of human-induced pluripotent stem cell- and embryonic stem cell-derived neural precursor cells (hPSC-NPCs) in 45-day culture period (Murphy et al., 2020).

Luo et al. developed surfactant-free and solvent-free PolyHIPEs and showed the proliferation of fibroblasts and cardiac muscle cells on PVA PolyHIPE hydrogels (Luo et al., 2015b). Recently, we showed cell viability and attachment of human dermal fibroblasts (HDFs) on PCL PolyHIPEs in comparison with commercially available styrene PolyHIPE scaffold (Aldemir Dikici et al., 2019b). SEM images of the HDFs suggested that the pore size of the PolyHIPEs have a profound effect on the orientation of the cells.

Moglia et al. developed injectable PCL PolyMIPEs with 20–200 KPa and 4–60 KPa compressive moduli and strengths, respectively. They suggested their use for soft tissue regeneration and showed the initial cytocompatibility of PolyHIPEs with the activity of hMSC higher than 95% after 72 h (Moglia et al., 2014b).

#### Drug-Releasing PolyHIPEs

Controlled release of drugs and bioactive molecules is desired for accelerating tissue regeneration, controlling biological responses or inhibiting pathology. PolyHIPEs are good candidates to elute drugs in a controlled manner as the surface area of these matrices can be precisely engineered. However, there is only a limited number of studies reported the PolyHIPE matrices as drug delivery tools.

Yang et al. (2017) incorporated enrofloxacin (ENR) solution (in DCM), a veterinary wide-spectrum antibiotic, into the oil phase of the PCL HIPEs. They also showed the possibility of fabricating scaffolds using two different inks (drug-loaded and non-loaded) for the selective construction of drug-loaded parts (Figure 12C). Drug-loaded PolyHIPEs showed a rapid release profile with 80 and 98% release in 2.5 and 10 h, respectively. Hu et al. dissolved ibuprofen, an anti-inflammatory drug, in the oil phase of PCL HIPE to create ibuprofen releasing PolyHIPE scaffolds (Hu et al., 2019). Burst release of the drug (75–90% for various compositions) was observed within the first 8 h. The release profile has been shown to be controllable by changing the concentration of the PCL. More research on the development of ibuprofen-loaded PCL (Hu et al., 2016), PLGA (Hu et al., 2014a), and PLA (Hu et al., 2014b, 2016) Poly(HIPEs/MIPEs) has been reported by the same group. They also incorporated bovine serum albumin (BSA) into the water phase of the HA stabilised Pickering emulsions and showed that the release profile of BSA could be controlled by changing HA concentration in the composition (Hu et al., 2016).

All of these studies suggested promising results for the use of PolyHIPEs in drug delivery applications. The common characteristics of all the studies mentioned above were the inclusion of the drugs in the emulsion composition before emulsification, and the use of toxic solvents in the emulsion composition to dissolve the polymers. Although scaffolds were left under vacuum to remove the solvent after solidification, they did not include any washing step for the removal of any leftover uncured materials or solvent as this step may also cause washout of the high amount of drugs from the scaffolds.

Moglia et al. developed bone morphogenetic protein 2 (BMP-2) releasing solvent-free ethylene glycol dimethacrylate PolyHIPE microspheres using w/o/w double emulsion system (Moglia et al., 2014a). They reported that while the encapsulation efficiency of their system was up to 73%, but this efficiency reduced to as low as 15% in the processes which require purification. In the follow-up study from the same group, they have shown the sustained release of BMP-2 in at least 14 days, and the retention of bioactivity was confirmed by osteogenic differentiation of osteoblasts cultured on these microspheres (Whitely et al., 2019).

## Where Are We Currently?

To date, PolyHIPEs based on a wide variety of synthetic and natural materials have been developed, characterised, and tested *in vitro*. It is beyond doubt that we have gained a greater understanding of this formulation technique over the last decade. In addition to producing favourable 3D porosity, the development of surface functionalisation methods have further improved cell-material interaction of the emulsion templated matrices and increased the potential of PolyHIPEs to be used in the medical industry.

This extensively tunable fabrication technique has been used for the manufacturing of TE scaffolds for various soft and hard tissues so far. The emulsion templated scaffolds have been demonstrated to support the *in vitro* growth of various cell types (Figures 13A–K); fibroblasts Johnson et al., 2015; Aldemir Dikici et al., 2019b), bone cells (Sherborne et al., 2018; Aldemir Dikici et al., 2019a, 2020), mesenchymal stem cells (Moglia et al., 2014b), mesenchymal progenitors (Owen et al., 2016; Aldemir Dikici et al., 2020), endothelial cells (Dikici et al., 2020a), neuronal cells (Barbetta et al., 2005a; Murphy et al., 2017, 2020), cardiac muscle cells (Luo et al., 2015a), and chondrocytes (Naranda et al., 2016). Although aforementioned *in vitro* results are promising, *in vivo* evaluation of the PolyHIPEs remains limited to the chick chorioallantoic membrane (CAM) assay which is a rapid and inexpensive *in vivo* platform to investigate initial tissue response to biomaterials and angiogenic agents (Dikici et al., 2019, 2020b; Mangir et al., 2019b). We previously reported testing of *in vivo* biocompatibility and angiogenic potential of PCL PolyHIPEs using an ex-ovo CAM assay (Figures 13L–O; Aldemir Dikici et al., 2019a, 2020).

Bringing medical devices to market is challenging in many countries due to the strict regulations on the commercialisation process (Bertram et al., 2012). For commercialisation and clinical use of PolyHIPE matrices, there are still many issues that need to be investigated, such as; the long-term behaviour of PolyHIPEs *in vivo* and their clinical validation, the evaluation of the integration of them with host tissue, how their mechanical properties are changing by time, sterilisation routes, and shelf life of these matrices (Plagnol et al., 2009).

One of the most important changes in the Medical Device Regulations (MDR) that come into force on May 2020 is that the human originated cells and tissues or their derivatives (in the same way as those of animal originated) will also be considered as a high-risk medical device (Class III) (Medical Device Regulation [MDR], 2020). Due to these regulatory restrictions, human or animal-sourced medical devices and implants will likely to have more restrictive approval processes and a more challenging pathway for clinical approval (Maak and Wylie, 2016; Hammerl et al., 2019; Haugen et al., 2019). Thus, synthetic source PolyHIPE matrices, in particular, are promising alternative substrates to be used for the fabrication of medical devices.

## Conclusion and Prospective Outlook

Emulsion templating is a favourable scaffold fabrication technique with various advantages, such as enabling high porosity, providing high interconnectivity, having high tunability of the architecture, mechanical properties and functionality, being suitable to be fabricated in various forms using a wide range of materials. Important to note is that emulsion templating can be used as a reliable fabrication method, but the production is dependent on a large number of process variables, and the fabrication setup is extremely sensitive to changes in the composition and condition of the process. Thus, to be able to have control over the morphology and the mechanical properties of the scaffolds, it is important to know the effect of individual parameters on the PolyHIPE properties. We devised this review as an update on the state-of-the-art of emulsion templating in TE and as a guide text for the use of emulsion templating as a TE scaffold fabrication route by summarising the key points that should be considered during the fabrication process of PolyHIPEs.

The main challenge of emulsion templating is to remove the toxic organic solvents used in emulsion composition and other impurities such as unreacted monomers and residual surfactant. Thus, especially solvent-free and surfactant-free HIPE compositions are considered as promising and cost-effective as they eliminate the solvent and impurity removal steps.

For improved scaffold-biological tissue interaction, more studies focusing on the development of o/w PolyHIPEs with enhanced mechanical properties and development functionalised w/o PolyHIPEs is needed. We are confident that emulsion templating will become an increasingly popular scaffold manufacturing technique in the next decade by considering the increasing number of publications on emulsions templating in TE. Also, future studies that concentrate on the investigation of long term behaviour of PolyHIPE matrices *in vivo* would aid to establish a greater degree of understanding on the potential of emulsion templated matrices to be used in the clinic.

## Author Contributions

BA performed the review and wrote the manuscript. FC provided feedback and edited the manuscript. Both authors contributed to the article and approved the submitted version.

## Conflict of Interest

The authors declare that the research was conducted in the absence of any commercial or financial relationships that could be construed as a potential conflict of interest.
